# Docking and quantitative structure–activity relationship of bi-cyclic heteroaromatic pyridazinone and pyrazolone derivatives as phosphodiesterase 3A (PDE3A) inhibitors

**DOI:** 10.1371/journal.pone.0189213

**Published:** 2017-12-07

**Authors:** Camila Muñoz-Gutiérrez, Daniela Cáceres-Rojas, Francisco Adasme-Carreño, Iván Palomo, Eduardo Fuentes, Julio Caballero

**Affiliations:** 1 Centro de Bioinformática y Simulación Molecular (CBSM), Universidad de Talca, Talca, Chile; 2 Platelet Research Laboratory, Department of Clinical Biochemistry and Immunohematology, Faculty of Health Sciences, Interdisciplinary Excellence Research Program on Healthy Aging (PIEI-ES), Universidad de Talca, Talca, Chile; 3 Núcleo Científico Multidisciplinario, Universidad de Talca, Talca, Chile; University of Parma, ITALY

## Abstract

PDE3s belong to the phosphodiesterases family, where the PDE3A isoform is the major subtype in platelets involved in the cAMP regulation pathway of platelet aggregation. PDE3A inhibitors have been designed as potential antiplatelet agents. In this work, a homology model of PDE3A was developed and used to obtain the binding modes of bicyclic heteroaromatic pyridazinones and pyrazolones. Most of the studied compounds adopted similar orientations within the PDE3A active site, establishing hydrogen bonds with catalytic amino acids. Besides, the structure-activity relationship of the studied inhibitors was described by using a field-based 3D-QSAR method. Different structure alignment strategies were employed, including template-based and docking-based alignments. Adequate correlation models were obtained according to internal and external validations. In general, QSAR models revealed that steric and hydrophobic fields describe the different inhibitory activities of the compounds, where the hydrogen bond donor and acceptor fields have minor contributions. It should be stressed that structural elements of PDE3A inhibitors are reported here, through descriptions of their binding interactions and their differential affinities. In this sense, the present results could be useful in the future design of more specific and potent PDE3A inhibitors that may be used for the treatment of cardiovascular diseases.

## Introduction

Phosphodiesterases (PDEs) are enzymes which degrade cAMP and/or cyclic guanosine monophosphate (cGMP).[1–3] They are classified into 12 PDE families, where several of them are cAMP-specific enzymes, others are cGMP-specific enzymes, and others use both cyclic nucleotides as substrate.[4] In particular, platelets contain three classes of PDEs: PDE2, PDE3, and PDE5. PDE3, which hydrolyzes both cAMP and cGMP, is the most abundant PDE in platelets. It exhibits low *K*_m_ for cAMP (0.2–0.5 μM) and is competitively inhibited by cGMP.

Cilostazol, cilostamide, enoximone, imazodan, and milrinone[2] were discovered as specific inhibitors of the PDE3 isoform and have demonstrated to serve as potent antiplatelet agents.[5] Platelets play a significant role in hemostatic and thrombotic processes, where abnormal platelet adhesion/activation can lead to the formation of clots (thrombosis).[6] Platelet function is modulated by many different agents, where the second messenger cAMP is a potent inhibitor of platelet activation. Intracellular cAMP levels are controlled through its synthesis rate by adenylate cyclase and/or its hydrolysis by PDE3 and PDE2 [7,8]. Ten years ago, Sun *et al*. observed that PDE3A was the primary subtype of PDE3 expressed in platelets.[9] As essential regulators of cyclic nucleotide signaling with diverse physiological functions, including inhibition of platelet aggregation, PDE3A has become recognized as an important drug target for the treatment of various diseases, such as heart failure, depression, asthma, inflammation and erectile dysfunction.[4,10–12]

Protein crystallographic structures of several PDEs are available in Protein Data Bank (PDB), including PDE3B. However, PDE3A structure is not available, but it shares a 69% of sequence identity with its homologous PDE3B isoform. This sequence identity makes it easy to construct a PDE3A model, which could be used for studying how inhibitors bind to its active site. Such study could be relevant for designing novel potent inhibitors. With this in mind, we constructed a three-dimensional (3D) molecular model of the PDE3A catalytic portion based on PDE3B’s X-ray crystal structure[13] in this work and performed molecular docking experiments to predict the binding modes of PDE3A inhibitors (bicyclic heteroaromatic pyridazinones and bicyclic heteroaromatic-pyrazolones) inside the developed 3D model.[14–18] After this, different correlation field-based 3D-QSAR models were built by using docked-based and template-based alignments. Results exposed here provide the basis for rational development of novel potent PDE3A inhibitors.

## Materials and methods

### Homology modeling

An available crystal structure of human PDE3B (code 1SO2 in the Protein Data Bank, 2.4 Å of resolution) [19] was used as a template for homology modeling of the PDE3A 3D-structure. This template was co-crystallized with an inhibitor containing a pyridazinone group (compound **14e** in reference [20]), denoted as PZO14e in this manuscript; this chemical group is a frequent moiety in most of the inhibitors currently studied. Query sequence of the human PDE3A catalytic domain (UniProt ID: Q14432, segment 728–1086) and PDE3B sequence share 66% identity.

The template structure was first prepared using Schrödinger’s Protein Preparation Wizard (PPW) software, including bond order assignment, hydrogen atoms addition, and protonation states prediction of the polar residues (Schrödinger Suite 2016–1 Protein Preparation Wizard; Epik, Schrödinger, LLC, New York, NY, 2016; Impact, Schrödinger, LLC, New York, NY, 2016; Prime, Schrödinger, LLC, New York, NY, 2016). Two Mg^2+^ ions, six water molecules which coordinate them, and the co-crystallized ligand were kept. Then, the system was subjected to molecular minimization using the Impact refinement module[21] and OPLS3 force field[22] with heavy atoms restrained to remain within a root-mean-square deviation (RMSD) of 0.30 Å from the initial coordinates.

Human PDE3A homology model was built using *Prime* from Schrödinger’s Suite (Prime, Schrödinger, LLC, New York, NY, 2016). The model secondary structure was predicted using the secondary structure prediction program (SSpro) bundled within Prime. The target (PDE3A) and template (PDE3B) sequences were aligned using the ClustralW method. The model structure was built using the energy-based method, keeping the ligand, magnesium ions and water molecules present in the structure. All structural discontinuities were modeled (template gaps greater than 20 residues are omitted by default), including two large loops spanning 17 residues (779–795) and 39 residues (1028–1066). An additional six-residue loop (923–928) was refined by minimizing a shell of 8.5 Å around this segment using the Prime Refinement module.

To relax the two unoptimized loops present in the model, we carried out a 10-ns restrained molecular dynamics (MD) simulation using the Desmond v.2.3 software[23] with the OPLS3 force field. The system was solvated into an orthorhombic box with a buffer distance of 10 Å, and neutralized by adding complementary ions. Simulation parameters were kept to their default values: ensemble NPT, constant temperature at 300 K using the Nosé-Hoover chain thermostat, and constant pressure at 1 atm using the Martyna-Tobias-Klein barostat, with relaxation times of 1 and 2 ps, respectively. RESPA integrator was applied with a time step of 2 fs for bonded and nonbonded-near forces, and 6 fs for long range forces. Particle-mesh Ewald was employed for long-range electrostatics with a cutoff radius of 9 Å. Protein backbone excluding the two loop regions, ligand, ions, and coordinated water molecules was restrained with a constant force of 5 kcal mol^-1^ A^-2^.

Afterward, we carried out a 50-ns MD simulation with the entire protein backbone and ions restrained with 0.5 and 5.0 kcal mol^-1^ A^-2^ constant forces, respectively. The ligand, magnesium ions and metal-coordination waters were unrestrained. This simulation aimed to equilibrate side chains, protein-ligand intermolecular interactions and hydrogen bond (H-bond) network within the active site. Simulation parameters were the same as in the previous MD simulation. Finally, ProSa[24] and PROCHECK[25] programs were used to assess the quality of the resulting molecular structure.

### Dataset preparation

**Fig 1:**
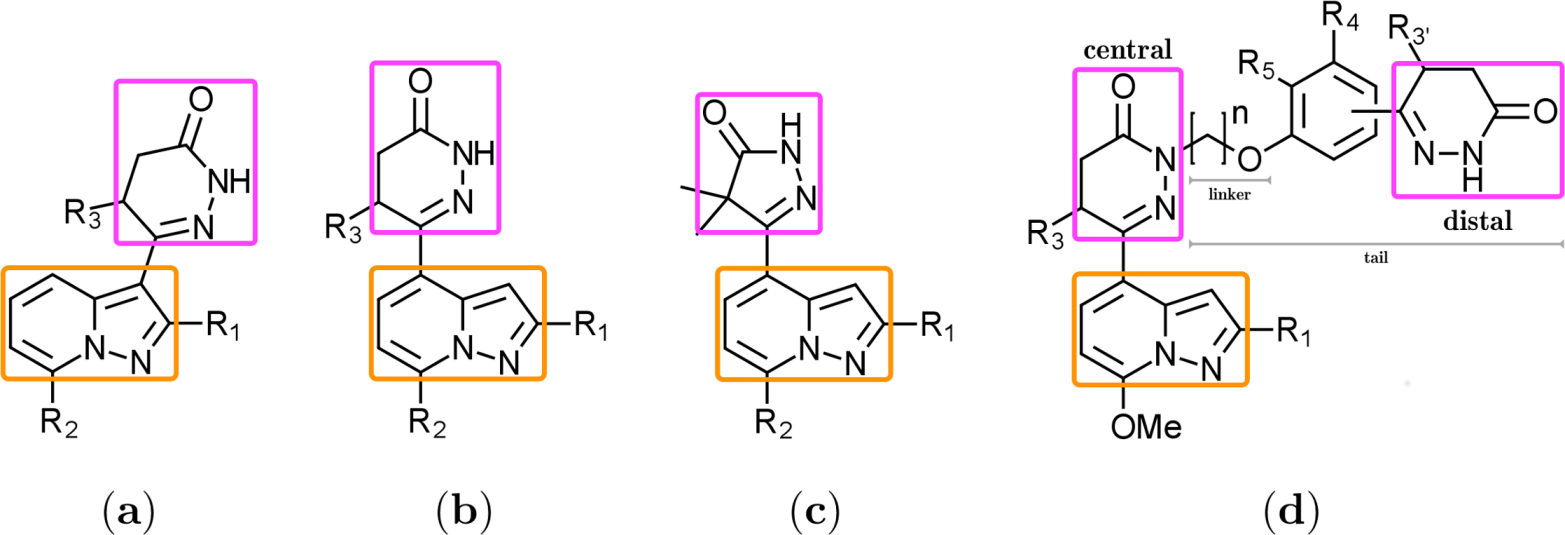
Structure of PDE3A inhibitor series. Common chemical scaffolds among the studied compounds are shown. Pyrazolopyridine and pyridazinone/pyrazolone moieties are highlighted in orange and magenta, respectively.

Five series of congeneric bicyclic PDE3A inhibitors (referred as sets **1**–**5**), composed by an heteroaromatic group (mainly pyrazolopyridine) and pyridazinone or pyrazolone, were extracted from references [14–18] (their general chemical structures are depicted in Fig 1). This data collection yielded a total of 107 compounds with reported inhibitory activities as IC_50_ ranging from 0.00027 to 400 μM. IC_50_ values were converted into logarithmic values log(10^6^/IC_50_) prior QSAR models’ elaboration. The distribution of the logarithmic activity values is shown in Figure A in S1 File. It can be observed that the data values follow a Gaussian distribution, concentrated in the micro Molar range (values between 5.0 and 6.0), indicating that the compounds considered in this work encompasses a high activity variety.

All compounds and their respective activities are listed in Table A in S1 File. Structures were sketched using Maestro’s molecular editor (Maestro, Schrödinger, LLC, New York, NY, 2016) and then prepared with LigPrep module (LigPrep, Schrödinger, LLC, New York, NY, 2016), where ionization states were generated at pH 7.0 ± 2.0 using Epik.[26] Energy minimization in the gas phase using Macromodel (MacroModel, Schrödinger, LLC, New York, NY, 2016) with the OPLS3 force field was performed during each ligand preparation. Compounds containing the pyridazinone ring have two possible enantiomers; the *R* enantiomer at the chiral center in the pyridazinone ring was chosen since it has the same chirality as the PZO14e compound in the PDE3B crystal structure.

### Molecular docking

All molecular docking calculations were performed using the Glide program[27,28] with the Standard Precision (SP) algorithm. Docking grids were generated with default settings using the co-crystallized ligand in the active site as centroid while ensuring that the grid box size was big enough to cover the entire active site. Default docking parameters were used enabling the option for enhancing planarity of conjugated π groups and including aromatic carbons as H-bond donors. All docking poses were visually inspected; filtering out those which did not establish analog interactions to the co-crystallized inhibitor, namely, H-bond with His961 and Gln1001, and a π-stacking interaction with Phe1004. These interactions were defined as the Essential Chemical Interactions Described for Analog Ligands (ECIDALs) for the PDE3A inhibitors that contain pyridazinone or pyrazolone.[29] In a few cases, core constraints with an RMSD tolerance of 1–2 Å regarding the pyridazinone ring position in the co-crystalized ligand was required to obtain docking poses which comply with these ECIDALs. Strain correction terms were applied during docking scoring. Best docking pose per compound was selected according to compliance with ECIDALs and lower *E*_model_ energy.

### QSAR modeling

QSAR models were computed to describe the structure-activity relationship of the PDE3A inhibitory activities. Those compounds for which the molecular docking did not yield satisfactory binding poses were excluded from this analysis. Thus, QSAR dataset comprised 99 randomly divided molecules into training and external test sets in a size ratio of 4:1; *i*.*e*., 79 and 20 compounds, respectively. Compounds were arranged by activity into 20 groups to ensure that the test set spanned the entire activity range, randomly assigning one compound to the test set for each group, whereas the remaining compounds were assigned to the training set. The distribution of the training and test set logarithmic activities is shown in Figure A in S1 File, where the values are similarly distributed in both sets, resembling the complete set, displaying a good sample of the entire activity range for QSAR modeling.

Alignment rule states that the molecular structures positioning within a lattice is a crucial input for all QSAR models.[30] We tested three aligning ways: (i) selecting the heteroaromatic group (mainly pyrazolopyridine, orange group in Fig 1) as a template, named pyrazolopyridine alignment (PPA) in this manuscript, (ii) selecting the amide ring moiety (pyridazinone or pyrazolone moiety, magenta group in Fig 1) as a template, named pyridazinone/pyrazolone alignment (PA) in this manuscript, and (iii) using the resulting structures from the molecular docking, effectively considering the ligand arrangement within the PDE3A active site, named docking alignment (DA) in this manuscript.

We generated field-based 3D-QSAR (FQSAR) models for the conformations in each alignment scheme. Three final FQSAR models were produced using PPA, PA and DA schemes. These FQSAR models were calculated using the Phase software (Phase, Schrödinger, LLC, New York, NY, 2016) as an implementation of the CoMFA[31] and CoMSIA[32] methods with a particular set of parameters. Lennard-Jones steric potentials and atomic charges for the electrostatic fields were taken and generated with the OPLS_2005 force field.[33,34] Hydrophobic fields were based on the atom types and hydrophobic parameters reported by Ghose *et al*.[35] H-bond acceptor and donor fields were based on Phase pharmacophore feature definitions, with projected points. FQSAR used a 30 kcal mol^-1^ threshold for both, van der Waals and electrostatic interactions, besides it eliminated grid points located too close to training set atoms. Additionally, before performing the Partial Least Squares (PLS) regression, fields in FQSAR were scaled by the standard deviation over the entire training set.

Fields were calculated on a rectangular grid enclosing the training set molecules. The grid spacing was set to 1 Å and the grid was extended 3 Å beyond the limits of the training set molecules. Grid points closer than 2 Å to any atom in the training set were excluded. Then, grid locations were used in a PLS fitting procedure with a maximum number of 6 PLS factors. Variables with a standard deviation less than 0.01 were eliminated.

The obtained 3D-QSAR models were evaluated using both internal and external validation. Internal validation involved the leave-one-out (LOO) cross-validation procedure ($R_{CV}^{2}$ in this text) and the stability estimate. The latter is computed as the *R*^2^ value between the LOO predictions and those obtained from the full training set. If the stability is high, the model is insensitive to changes in the training set. For external validation, root-mean-square errors (RMSE) and *R*^2^ value of the test set ($R_{test}^{2}$ in this text) were employed. Models were acceptable if $R_{CV}^{2}>0.5$ and $R_{test}^{2}>0.5$, as these criteria are indicative of reliable and predictive models.[36,37] Best models were those with high $R_{CV}^{2}$, $R_{test}^{2}$ and stability values, as well as low RMSE values.

## Results and discussion

### Protein modeling

**Fig 2:**
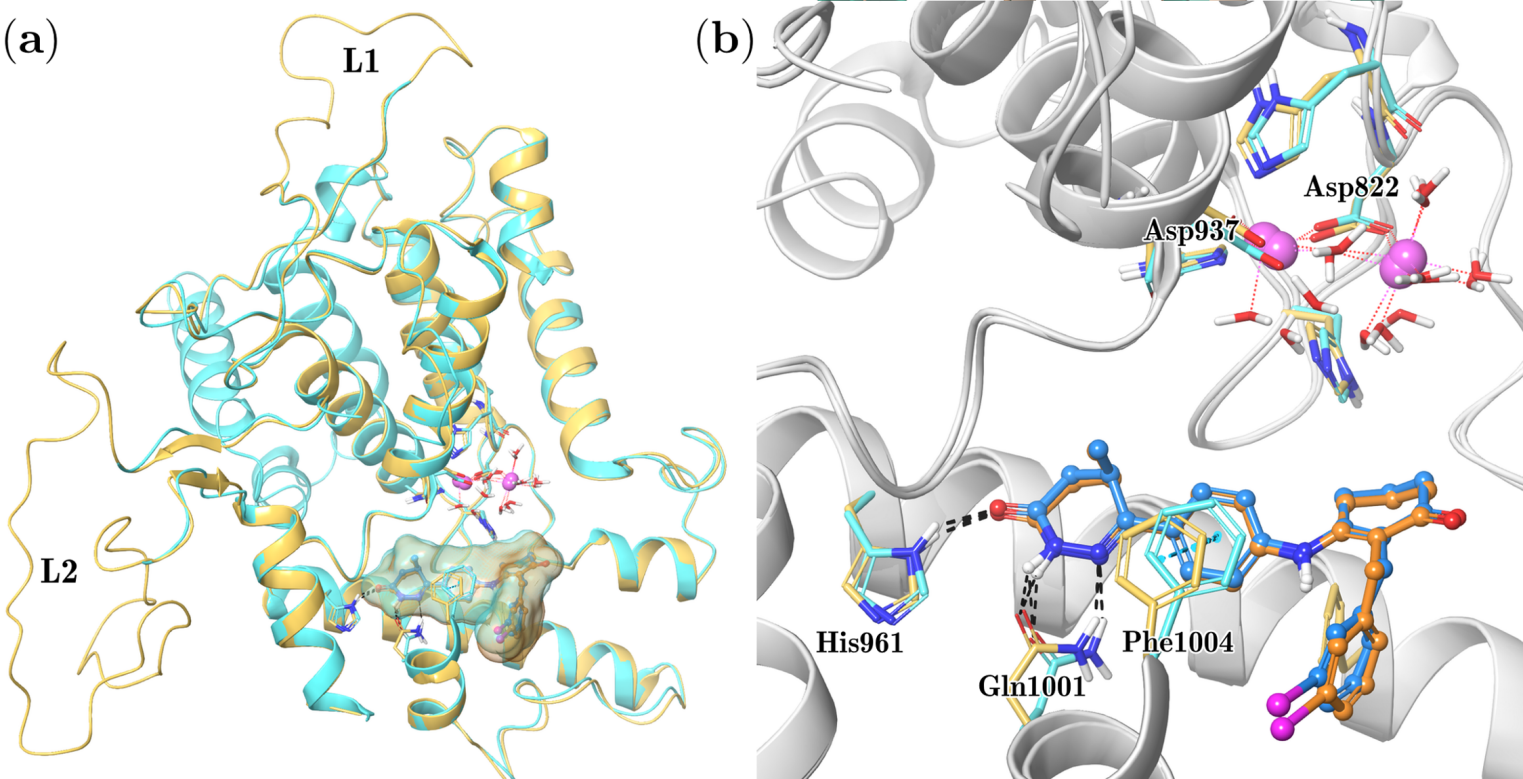
Superposition of PDE3A model (pale orange) to the PDE3B crystal structure (cyan). (a) Protein structure visualization presenting modeled loops (L1 and L2), and the location of metal and ligand binding site. (b) Comparison of PDE 3A/3B active sites; tertiary protein structure is shown in ribbons, relevant protein residues and water molecules are shown as thick tubes, magnesium ions are shown as van der Waals spheres, and ligands are shown in ball-and-stick representation; dotted lines indicate zero-order bonds, dashed black lines represent H-bonds, and cyan dashed lines indicate π-stacking interactions. Carbon coloring are: cyan and light blue for protein and ligand atoms in the PDE3B crystal, and pale orange and orange for protein and ligand atoms in the PDE3A model.

We modeled PDE3A catalytic domain since there is not an available crystallographic structure at the time of this work. We employed the PDE3B crystal structure co-crystallized with the PZO14e compound as a template [19]. The high sequence identity (~65%) between these proteins suggests that the structure modeled would be accurate, as homology models with a sequence identity above 50% tend to be reliable, with only minor errors in the side chains.[38] The resulting PDE3A model, presented in Fig 2, shows that secondary structure elements of PDE3B, mainly α-helices, are conserved in PDE3A. Two large regions missing in the PDE3B crystal, residues 779–795 and 1028–1066 were refined via restrained MD simulations; they are shown in Fig 2A as L1 and L2, respectively. The final protein model was first assessed with ProSa,[24] where the model and the reference crystal (PDB code 1SO2) shared very similar Z-scores: -8.41 and -8.39, respectively. The stereochemical quality was evaluated via PROCHECK,[25] where the model structure showed only one residue (0.3%) in disallowed regions. Altogether, these estimates indicated the proper quality of the predicted structure.

An additional MD simulation was performed to relax the PDE3B model binding site. Sequence alignment showed that residues forming the active site are highly conserved in the 3A/3B subtypes. The dihydropyridazinone ligand PZO14e co-crystallized inside the PDE3B template also has inhibitory activity against PDE3A (compound **14e** in reference [20]). Since the dihydropyridazinone group is present in the compounds under study (the magenta group in Fig 1), we considered that interactions of this group should be similar in our compounds. Thus, the additional PDE3A MD simulation was performed in the presence of PZO14e, which forms H-bonds with residues His961 and Gln1001 and π-stacking with the Phe1004 side chain. Protein fluctuation and other relevant molecular structures during this MD are presented in Figure B in S1 File. It was evidenced that the overall protein tertiary structure was stable after 30-ns MD simulation. Both ligand and magnesium ions displayed only minor movements along the trajectory, whereas metal-coordination waters required a 15-ns period for stabilization.

We also monitored interactions between the PZO14e dihydropyridazinone group and His961, Gln1001, and Phe1004 residues along the MD trajectory (see Figure C in S1 File). The intermolecular interactions exhibited fluctuations ≤0.25 Å, where the H-bond formed between the side chain amine group of Gln1001 and the unprotonated amine of the dihydropyridazinone was the least stable. In general, active site residues forming H-bonds, *i*.*e*., magnesium ions, metal-coordination waters, and the PZO14e ligand; exhibited minor movements, indicating the overall stability of the model structure. PZO14e position regarding these chemical features is represented in Fig 2B; it is important to note that the ligand is not in direct interaction with metal ions. Developed PDE3A model coordinates are included in Suplementary material (PDE3A_model.pdb).

### Prediction of the binding modes

Prior docking of the PDE3A inhibitors under study, we assessed the ability of docking methodology to reproduce the crystal conformation by predicting the PZO14e binding mode within the PDE3B crystal. This evaluation yielded an RMSD of 0.48 Å for the pyridazinone-phenyl substructure, but the hexadione-phenyl moiety was placed in a different orientation. However, the latter group lies in a wide solvent-exposed pocket with no evident protein-ligand interaction in the crystal that may serve as anchoring point.

There is no available structural information about the binding mode of pyrazolopyridine-pyridazinone/pyrazolone derivatives (compounds in sets **1**–**5**) within the PDE3A active site. However, considering that the PDE3A inhibitors share the pyridazinone-phenyl PZO14e substructure, it can be expected that they would bind in a similar arrangement to this compound, establishing intermolecular interactions with the residues His961, Gln1001 and Phe1004 (defined as ECIDALs). Dihydropyridazinone compound zardaverine was crystallized bound to PDE4D[39] exhibiting a different orientation than PZO14e, where the pyridazinone ring is interacting with the metal binding site. However, various residues in PDE4D would prevent the ligand to be placed like PZO14e, *i*.*e*., Gly953Asn and His961Tyr. Moreover, zardaverine binding mode could not accommodate larger substitutions such as those present in the set inhibitors **5**. Consequently, the PZO14e binding conformation was used as reference for defining the ECIDALs in our study.

**Fig 3:**
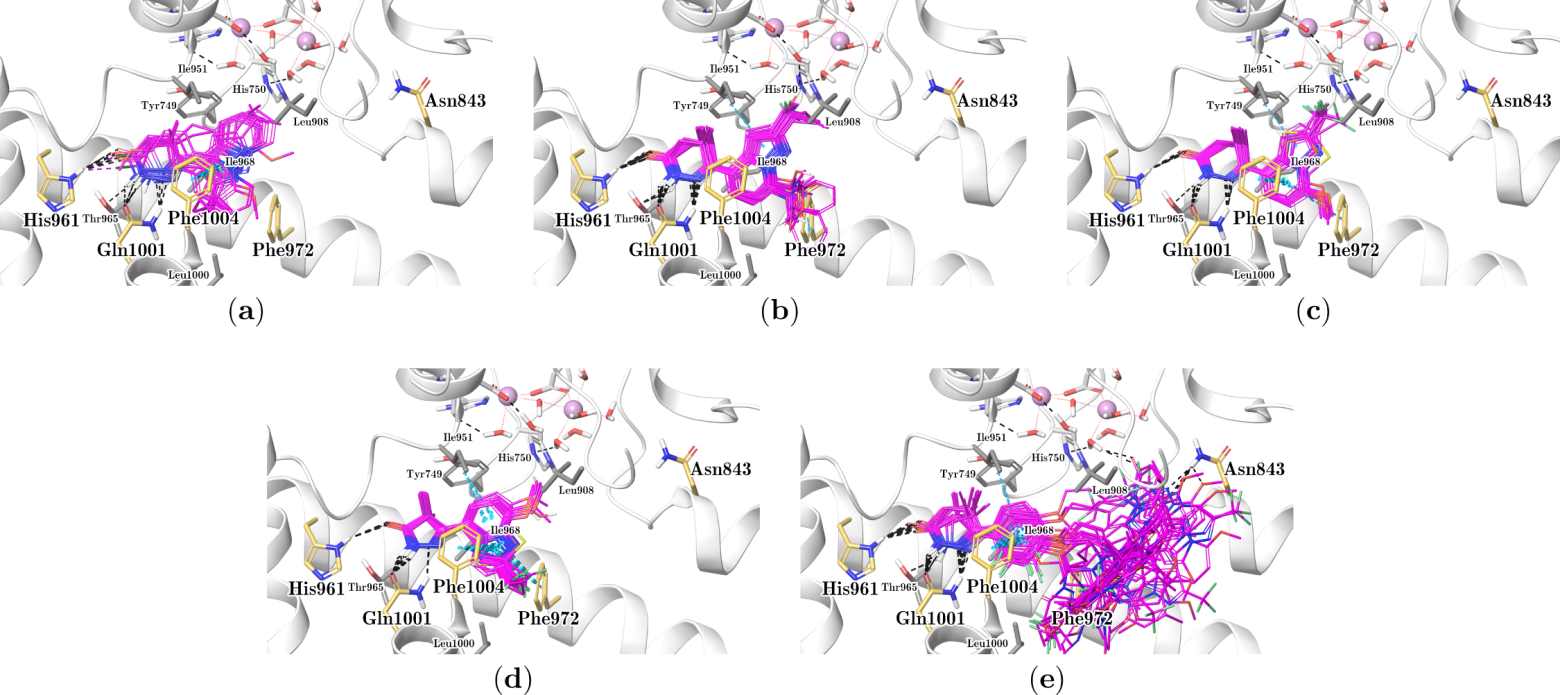
Predicted binding modes of the studied compounds within the PDE3A binding site. Each inhibitor series **1**–**5** is shown separately: set **1** in (a), set **2** in (b), set **3** in (c), set **4** in (d), and set **5** in (e). Ligands are shown in thin tubes representation with magenta carbons. Protein residues and metal-coordinating waters are displayed in thick tubes, and magnesium ions as van der Waals spheres. PDE3A residues are shown in gray, ligand binding residues in pale orange, and metal binding residues in light gray. Dashed black lines indicate H-bonds and cyan dashed lines denote π-stacking interactions. The tertiary structure is shown in white ribbons.

Fig 3 shows that the majority of docked compounds adopted approximately the same position within the active site. Most of them spontaneously comply with the defined ECIDALs during docking process; however, some inhibitors yielded an orientation that complies with ECIDALs after applying distance constraints. Eight out of the 107 molecules did not yield satisfactory docking poses, even using constraints: **1–14**, **1–16**, **1–17**, **1–18**, **1–31**, **1–43**, **1–45**, and **5-3l**. Most of them belong to set **1**, which contains the least active compounds of the entire series. Compounds **1–14**, **1–16**, **1–18**, **1–31**, **1–43**, and **1–45** have a benzyl group substituting the protonated amine of the pyridazinone scaffold, which prevents the interactions of these compounds with Gln1001. The other two inhibitors, **1–17** and **5-3l**, have some unfortunate substitution patterns which prevented them from fitting within the employed conformation of the active site. Considering that these molecules have inhibitory activity against PDE3A, albeit low, we argued that there should be significant induced fit effects upon these compounds binding, which was not modeled in this investigation. Given that these molecules did not generate docking poses, they were omitted from structure-activity relationship analyses.

Fig 3A presents docking poses for compounds in set **1** (12 in total). Such conformations exhibited a sigificant misalignment degree of the pyridazinone ring compared to the remaining inhibitors. This outcome is probably due to considerable changes in the pyridazinone ring, where covalent bonds fuse pyridazinone and pyrazolopyridine at different positions (*e*.*g*., **1–13**, **1–27**) producing bulkier ligand shapes, thus restraining the relative orientation that pyridazinone can take within the active site. This point would also justify the lower activity of this inhibitor set compared to the entire series. Despite this, most of the compounds in set **1** comply with the intermolecular interactions defined by ECIDALs. Table 1 summarizes the interaction averaged distances between chemical groups of the pyridazinone and the residues His961, Gln1001 and Phe1004 for Set **1**. All molecules established an H-bond with the Gln1001 amine group with an average distance of 3.21 ± 0.24 Å. However, only 9 out of the twelve also exhibited H-bonds with either the Gln1001 carbonyl group (2.99 ± 0.12 Å) or His961 (3.45 ± 0.17 Å), or both. In contrast, for the other three compounds of the set, the least active inhibitors **1–32**, **1–33** and **1–34**, did not exhibit H-bond with His961 because they have a carbonyl group substitution for the pyridazinone ring by either fluorine or methoxy (they contain a substituted pyridazine instead of pyridazinone). Accordingly, the pyridazinone group seems to be essential for establishing the most relevant interactions defined by ECIDALs, with striking effects on the activity of compounds in Set **1**. A π-stacking interaction with Phe1004 (4.15 ± 0.15 Å) was observed for most of these compounds.

**Table 1:** Averaged H-bond (first four columns) and π-stacking interaction (last two columns) distances for the ligand-PDE3A complexes extracted from molecular docking experiments.^a^.

Set	His961^*b*^		Asn843^*b*^		Gln1001^*b*^				Phe972^*c*^		Phe1004^*c*^	
					NH_2_		CO					
	*N*	*r*	*N*	*r*	*N*	*r*	*N*	*r*	*N*	*r*	*N*	*r*
1	9	3.45 ± 0.17			12	3.21 ± 0.24	7	2.99 ± 0.12			9	4.15 ± 0.15
2	33	3.45 ± 0.18			35	3.01 ± 0.05	31	2.99 ± 0.08				
3	8	3.46 ± 0.20			11	3.00 ± 0.03	11	3.00 ± 0.07	3	5.44 ± 0.05	7	3.53 ± 0.04
4	11	3.64 ± 0.04			14	3.12 ± 0.08	19	2.77 ± 0.06	19	5.35 ± 0.05	19	3.71 ± 0.11
5	20	3.34 ± 0.23	7	3.00 ± 0.11	22	3.10 ± 0.12	12	2.87 ± 0.18			20	3.90 ± 0.21

^*a*^*N* is the number of structures in which the interaction was found (*N* out of 12, 35, 11, 19 and 22 for sets **1**–**5**, respectively), and *r* is the observed average distance (in Å) in those structures.

^*b*^*r* is measured between the H-bond acceptor and donor atoms.

^*c*^Interaction where the planes of the aromatic rings are either in a sandwich or parallel-displaced conformation at a distance equal to or less than 5.5 Å.

*r* is measured between the center of the aromatic rings.

Compounds included in set **2** (35 in total) contain the 6-{pyrazolo[1,5-a]pyridin-4-yl}-2,3,4,5-tetrahydropyridazin-3-one scaffold (Fig 1B; see Table A in S1 File), with several substitutions at positions 2 and 7 (R_1_ and R_2_) of the pyrazolo[1,5-a]pyridine group, and a CH_3_ substituent at position 5 (R_3_) of the pyridazinone. Fig 3B presents the remining binding modes of compounds in set **2,** and Table 1 reports the averaged distances of the intermolecular interactions between the pyridazinone and the PDE3A residues. Most of the compounds in set **2** established the H-bonds between the pyridazinone and the residues His961 (averaged distance of 3.45 ± 0.18 Å) and Gln1001 (averaged distances of 3.01 ± 0.05 Å and 2.99 ± 0.08 Å with the side chain amine and carbonyl groups, respectively). Compounds **2-2l**, **2-2q**, **2-2z**, and **2-2ah** established an H-bond with the side chain OH of Thr965 instead of Gln1001; this residue is very close to the Gln1001 side chain with a possible contribution for the H-bond formation with the pyridazine group (it is similar in the crystallographic structure of PDE3B-PZO14e complex). Compounds **2-2e** and **2-2f** are correctly oriented, but they do not form the H-bond with His961. Unlike set **1**, compounds from set **2** did not establish the π-stacking interaction as the pyrazole ring was placed away from Phe1004. The methyl group at R_3_ of the pyridazinone ring is positioned at a tight hydrophobic cavity formed by Ile951 and Pro954. The pyrazolopyridine ring for compounds in the set **2** is placed in another hydrophobic region flanked by Tyr749, Leu908, Ile951, Ile968, Phe972, Leu1000, and Phe1004 (shown in Fig 3B). Substituents at position 2 (R_1_) of the pyrazolopyridine ring are oriented towards the metal binding site where they are located, in proximities of water molecules coordinated to the metals and His750 side chain. Nevertheless, shorter substituents (*e*.*g*., Me, Et) did not reach such region and rather are directed towards any of the nearby hydrophobic residues. Interestingly, R_1_ = H (compound **2-2v**) yielded the most active compound of the set **2** (IC_50_ = 0.06 μM), which is about one order of magnitude more potent than the closest analogue (compound **2-2ai**, R_1_ = CN, IC_50_ = 0.5 μM). Substituents at position 7 (R_2_) of the pyrazolopyridine face the solvent media; being surrounded by some hydrophobic residues. Therefore, it is assumed that such groups might have hydrophobic effects or affect ligand solubility.

Compounds included in the set **3** (11 in total) are analogous of compounds in set **2** with R_1_ = CF_3_ or Et, R_2_ = OCH_3_, R_3_ = CH_3_, and modify heterocyclic rings replacing pyrazolopyridine (Fig 1B; see Table A in S1 File). Fig 3C shows their obtained docking poses and Table 1 reports the averaged distances of the intermolecular interactions between their pyridazinone groups and the PDE3A residues. All compounds in set **3** create the H-bonds between the pyridazine amines and the side chain NH_2_ and CO groups of the residue Gln1001 (both averaged distances are 3.00 Å). Most compounds form H-bond with His961 (averaged distance is 3.46 ± 0.20 Å); only three compounds (*i*.*e*., **3–1**, **3–3** and **3–8**) lacked this H-bond with His961 as the pyrazolopyridine moiety moved away from such amino acid. π-stacking interactions with Phe1004 were identified for seven compounds in the set **3** (Table 1), and additional T-shaped π-stacking interaction with Phe972 (the averaged distance between the centers of mass of the aromatic rings is 5.44 ± 0.05 Å) was identified for some inhibitors of this set.

Compounds included in set **4** (19 in total) contain pyrazolone instead of pyridazinone (Fig 1C; see Table A in S1 File), featuring similar substitutions to those in set **2** at the pyrazolopyridine ring. The analysis of this set activities suggests that the altered scaffold did not yield a significant gain in inhibitory potency compared to compounds in sets **2** and **3**. Fig 3D shows docking poses for compounds in set **4** and Table 1 reports the averaged distances of the intermolecular interactions between pyrazolone groups and the PDE3A residues. The pyrazolopyridine ring for compounds in the set **4** had a different orientation (it was flipped when compared with the binding modes of compounds from sets **2** and **3**). The pyrazolone ring, smaller than pyridazinone, is more strained and is reflected in the intermolecular interactions with the PDE3A residues; 11 out of the 19 compounds formed the H-bond with His961 with slightly longer averaged distance (3.64 ± 0.04 Å), and five inhibitors did not establish the H-bond with the side chain NH_2_ of Gln1001. However, all compounds of this set formed H-bond with the side chain CO of Gln1001 and their pyrazole rings are placed between Phe972 and Phe1004 establishing π-stacking interactions with both amino acids.

Compounds included in set **5** (23 in total) are the most active among the PDE3A inhibitors in this study. They have the pyridazinone-pyrazolopyridine scaffold from set **2** and an additional distal pyridazinone separated from the first one through a phenoxyalkyl linker (Fig 1D; see Table A in S1 File). Distal pyridazinone contains all the polar groups available to form ECIDALs interactions, but the pyridazinone at the pyridazinone-pyrazolopyridine scaffold has a substitution at the pyridazine protonated NH. Obtained docking poses for compounds in set **5** (shown in Fig 3E) display the distal pyridazinone positioned within the binding pocket, near residues His961 and Gln1001. Table 1 reports average distances of the intermolecular interactions between the distal pyridazinone groups and PDE3A residues. Most compounds in set 5 formed the H-bond with His961 (averaged distance of 3.34 ± 0.23 Å), the H-bond with the side chain amine group of Gln1001 (averaged distance of 3.10 ± 0.12 Å), and the π-stacking interaction with Phe1004 (averaged distance of 3.90 ± 0.21 Å). About half of the compounds do not form the H-bond with the side chain CO of Gln1001, but establish the H-bond with the side chain OH of Thr965, which is very close to Gln1001. The pyridazinone-pyrazolopyridine compounds scaffold from set **5** (Fig 1D) is positioned differently for each ligand in different hydrophobic regions of the binding site entrance. For some compounds, either of these rings was placed below the metal binding site forming H-bond with the Asn843 side chain amine or metal-coordinating waters.

### QSAR models

**Fig 4:**
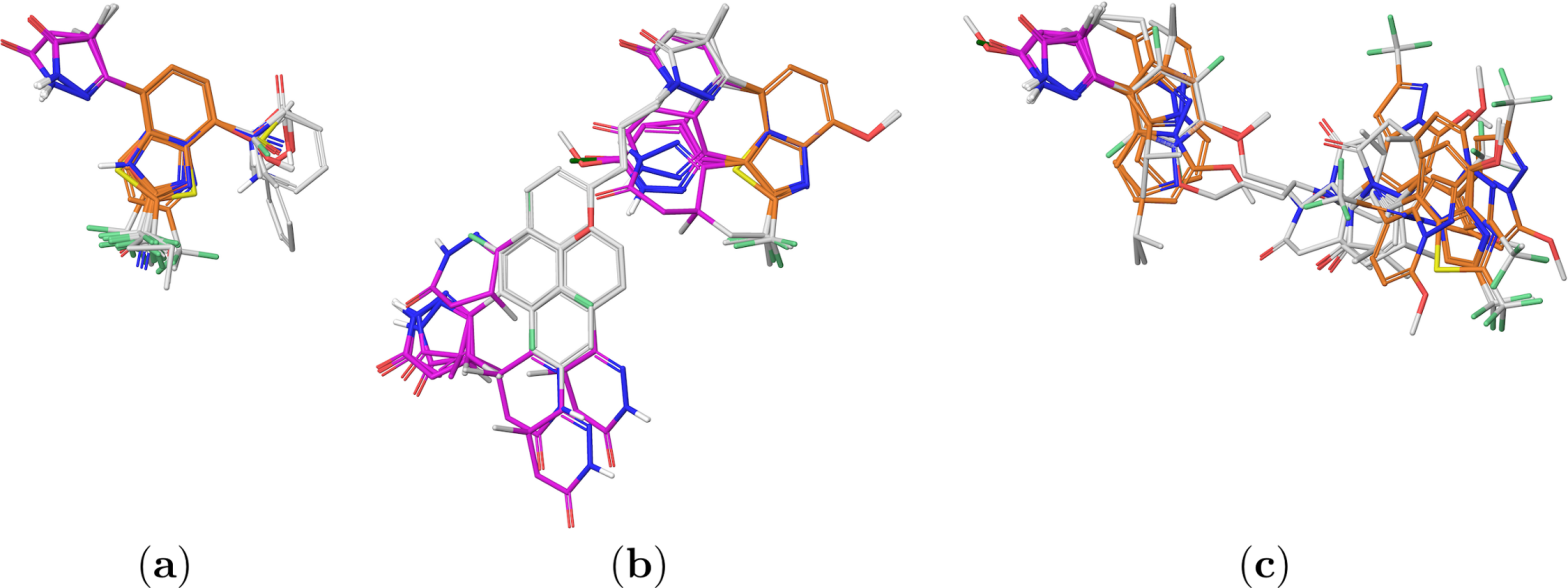
Template-based alignments of PDE3A inhibitors. (a) Sets **2–4**, (b) sets **1** and **5** in alignment PPA, and (c) sets **1** and **5** in PA. Pyrazolopyridine and pyridazinone/pyrazolone moieties are highlighted in orange and magenta, respectively.

We computed FQSAR models to understand the structure-activity relationships of PDE3A inhibitors under study. Initially, the compounds were superimposed by chemical similarity using the largest basic structure as a template: the pyrazolopyridine moiety and analog heterocyclic groups (Fig 1, orange), defined as pyrazolopyridine alignment (PPA) in this text. However, the PDE3A binding site as well as the binding modes obtained from the docking experiments indicate that the pyridazinone/pyrazolone moiety (Fig 1, magenta) establishes the prime interactions (defined as ECIDALs) with the PDE3A protein residues. Due to this information, we also tested another alignment using pyridazinone/pyrazolone as a template defined as pyridazinone/pyrazolone alignment (PA) in this text. Furthermore, we also examined the construction of FQSAR models based on the docked structures (defined as docking alignment; DA), since these 3D structures adequately represent the ligand conformations adopted within the binding site. Such alignment approach has led to the rigth predictions in previous QSAR investigations.[40,41] Fig 4 displays the resultant molecular alignments. Compounds from sets **2**–**4** have the same spatial arrangement in PPA and PA schemes (Fig 4A). Fig 4B and 4C respectively represent compounds alignments of sets **1** and **5** under PPA and PA schemes.

FQSAR models were derived from the three alignment strategies using different combinations up to five Gaussian fields: steric (*S*), electrostatic (*E*), hydrophobic (*H*), H-bond donor (*D*), and H-bond acceptor (*A*). To avoid over-fitting, up to six PLS factors were employed. Statistical criteria measuring the LOO cross-validation performance ($R_{CV}^{2}>0.5$) and test set predictions (i.e., $R_{test}^{2}>0.5$ and RMSE) were calculated to choose the most reliable and predictive models. The latter serves as a good estimate of the models’ applicability to predict the activity of novel compounds.[36,37] Table 2 lists the description and statistical information of the best FQSAR models.

**Table 2:** Statistical information of FQSAR models.^a^.

Alignment	Fields	NC	*R*^2^	$R_{CV}^{2}$	SD	Stability	$R_{test}^{2}$	RMSE
PPA	S	4	0.71	0.57	0.70	0.95	0.51	0.92
	E	6	0.85	0.61	0.51	0.86	0.69	0.74
	H	6	0.86	0.70	0.49	0.90	0.54	0.89
	A	5	0.68	0.53	0.74	0.92	0.66	0.77
	D	6	0.66	0.49	0.77	0.88	0.66	0.76
	HA	6	0.87	0.71	0.47	0.91	0.61	0.82
	SHA	6	0.87	0.72	0.47	0.91	0.56	0.88
	**SHAD**	**6**	**0.88**	**0.72**	**0.45**	**0.90**	**0.58**	**0.86**
	SEHAD	6	0.89	0.71	0.44	0.90	0.58	0.85
PA	S	4	0.78	0.59	0.61	0.90	0.62	0.82
	E	2	0.69	0.52	0.71	0.95	0.50	0.93
	H	5	0.84	0.68	0.53	0.93	0.54	0.89
	A	5	0.68	0.55	0.74	0.95	0.53	0.90
	D	6	0.24	0.09	1.15	0.65	0.19	1.18
	EH	5	0.86	0.71	0.49	0.93	0.53	0.9
	SEH	6	0.87	0.73	0.47	0.93	0.51	0.92
	**SEHD**	**6**	**0.88**	**0.73**	**0.46**	**0.93**	**0.56**	**0.87**
	SEHAD	6	0.88	0.72	0.46	0.93	0.57	0.86
DA	S	1	0.5	0.42	0.88	0.99	0.47	0.96
	E	2	0.67	0.20	0.72	0.76	0.39	1.02
	**H**	**1**	**0.69**	**0.51**	**0.69**	**0.93**	**0.52**	**0.91**
	A	1	0.52	0.30	0.86	0.93	0.41	1.01
	D	3	0.39	0.22	0.99	0.92	0.08	1.26
	HD	2	0.78	0.57	0.59	0.92	0.41	1.01
	EHD	2	0.79	0.55	0.57	0.89	0.43	0.99
	EHAD	2	0.79	0.52	0.57	0.87	0.48	0.95
	SEHAD	3	0.82	0.50	0.54	0.81	0.44	0.99

^a^ The best models are represented in boldface.

NC is the number of PLS factors; SD is the standard deviation of the fitted activity of the training set; *R*^2^, $R_{CV}^{2}$ and $R_{test}^{2}$ are the coefficients of determination of the training set, leave-one-out cross validation, and test set, respectively; Stability is computed as the *R*^2^ between the leave-one-out and the full training set predictions; RMSE is the root-mean-square error of the test predictions. Fields are steric (S), electrostatic (E), hydrophobic (H), and H-bond acceptor (A) and donor (D).

**Fig 5:**
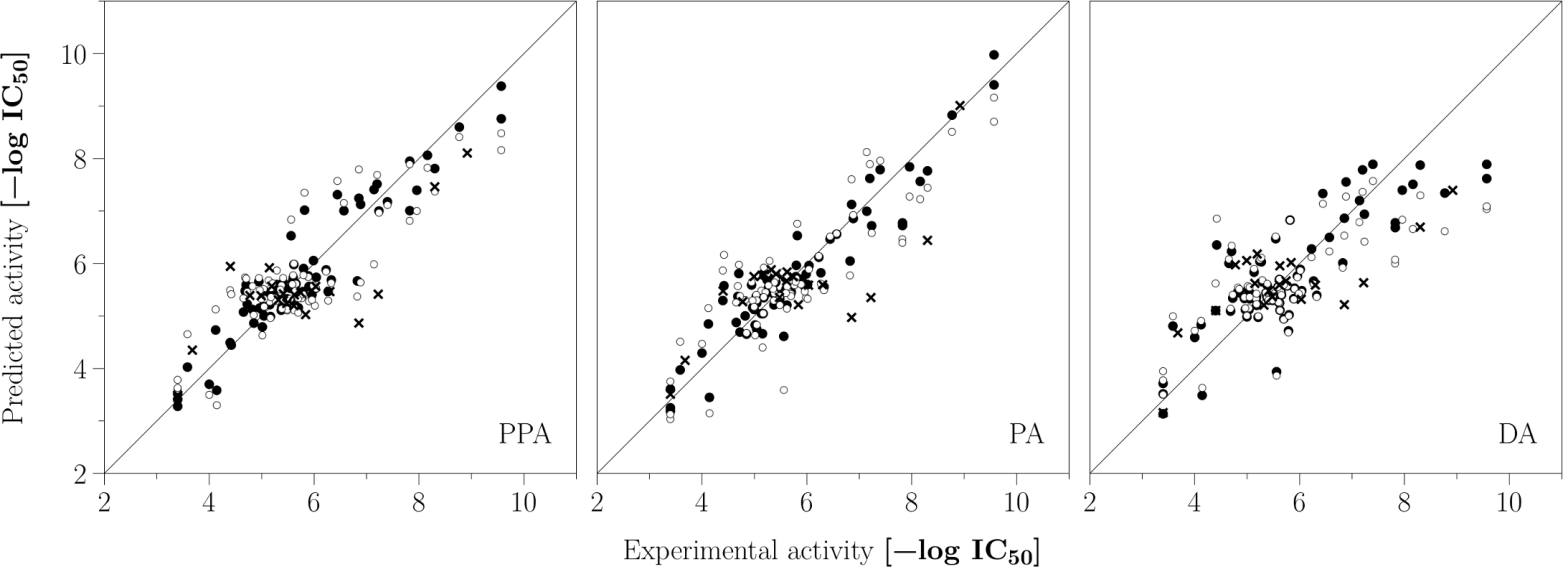
Scatter plots of the experimental vs. predicted activities for the best FQSAR models. Plots are shown for alignments PPA, PA, and DA. (●) training, (○) LOO cross-validated, (×) test set predictions, and solid line is for x = y.

Most of the FQSAR models were statistically adequate according to LOO validation ($R_{CV}^{2}>0.5$ and stability > 0.9) and external set predictive capacity ($R_{test}^{2}>0.5$). For PPA scheme, most of the models had acceptable performance using one of the five fields ($R_{CV}^{2}>0.5$ and $R_{test}^{2}>0.5$). Slightly better results were obtained by combining two or more fields. The best PPA-*SHAD* model includes steric, hydrophobic, H-bond acceptor and H-bond donor fields with a performance of $R_{CV}^{2}=0.72$ and $R_{test}^{2}=0.58$, using six components. This model has field contributions of 34.3% (*S*), 33.3% (*H*), 20.8% (*A*), and 11.6% (*D*), respectively. Fig 5A presents the experimental scatter plots against predicted activities (expressed as pIC_50_) for the training set, LOO cross-validation, and a test set for PPA scheme. It can be observed that PPA-*SHAD* model yielded successful predictions for the test set compounds.

For the PA scheme, the combination of four fields produced the best model PA-*SEHD* with similar performance for the best PPA model ($R_{CV}^{2}=0.73$ and $R_{test}^{2}=0.56$). The PA-*SEHD* model has a steric contribution of 45.2%, an electrostatic contribution of 14.5%, a hydrophobic contribution of 33.1%, and an H-bond donor contribution of 7.1%. Scatter plots of the experimental activities against predicted activities for the training set, LOO cross-validation, and test set for PA scheme, presented in Fig 5B, demonstrate that PA-*SEHD* model adequately describes the structure-activity relationship of the studied PDE3A inhibitors. It is noteworthy that in both PPA-*SHAD* and PA-*SEHD* models, the steric and hydrophobic fields have the major contributions, whereas the polar (electrostatic or H-bond) contributions are less significant in both schemes. This point suggests that the hydrophobic and steric effects mainly modulates the differential activities of the studied compounds.

In contrast, FQSAR results for DA scheme were rather poor, where only one model with the hydrophobic field was deemed as statistically adequate ($R_{CV}^{2}>0.5$ and $R_{test}^{2}>0.5$). These results are in agreement with previous reports where authors found that docking based alignments yield poorer QSAR predictive models since the fluctuations in the positions of common or analogous atoms due to different compounds conformations inside the binding site have a negative influence when generating fields.[41–43] The combination of multiple fields during FQSAR application under the DA scheme did not produce any substantial increase of the statistical performance. The DA-*H* model produced $R_{CV}^{2}=0.51$ and $R_{test}^{2}=0.52$ with one PLS component. Inspection of the scatter plot of the predicted activities presented in Fig 5C revealed that the calculated activities for compounds from set **5** (pIC_50_ > 7.0) substantially deviates from the diagonal, hindering the overall performance. Such outcome probably occurred due to the high flexibility of the pyrazolopyridine-pyridazinone/pyrazolone tail group within the solvent-exposed region of the binding site. Despite the above, errors for the test set predictions were only slightly greater than those calculated for previous models. As a matter of fact, the correlation plot (Fig 5C) shows that DA-*H* yielded comparable predictions of the test set compounds to those predicted by PPA-*SHAD* and PA-*SEHD*; however, predictions of the most active compounds were more accurate when using the template for PPA and PA schemes.

The residual plots of the predictions for the three selected FQSAR models are presented in Figure D in S1 File, and show that residual distributions were independent and random. A random residuals distribution in the corresponding training and test data suggests that the models fit the data well, where most values are below 1. In addition, 73%, 73%, and 52% of the studied compounds have residuals less than 0.5 for PPA, PA, and DA models, respectively. The mean signed errors were -0.04 for PPA, -0.02 for PA, and -0.03 for DA, and mean unsigned errors were 0.40 for PPA, 0.39 for PA, and 0.58 for DA, confirming poorer predictions for the DA model.

Altogether, QSAR results using a template and docking alignment schemes suggest that the structure-activity relationship of the PDE3A inhibitor series in this study can be primarily explained based on nonpolar chemical features. Also, correlation plots between experimental and predicted activities (–logIC_50_) presented in Fig 5 revealed that the best models discriminate the more active, from the less active inhibitors. Therefore, we propose that the selected FQSAR models can reasonably predict the order in the inhibitory activity of the studied compounds.

**Fig 6:**
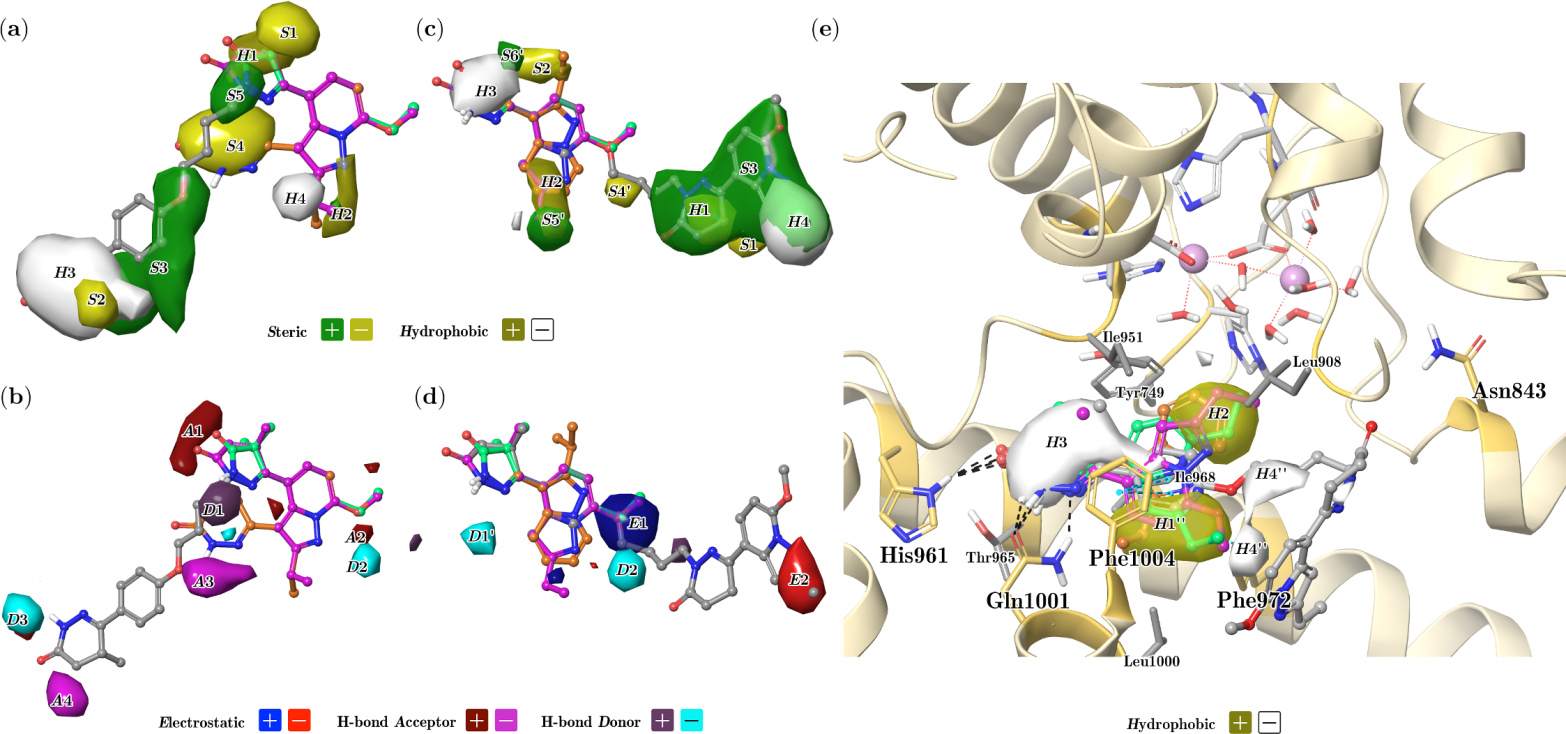
FQSAR contour maps of the best models of PDE3A inhibitors for the PPA (a, b), PA (c, d) and DA (e) schemes. Compounds **1–2** (orange), **2-2b** (purple), **4-6b** (light green), and **5-3q** (gray) are included as reference. In (e), the metal and ligand binding sites are shown for comparison following the same styling as Fig 3. Fields include steric (*S*), electrostatic (*E*), hydrophobic (*H*), H-bond acceptor (*A*) and donor (*D*). Images were generated using contour for positive and negative contribution values of 4.0 × 10^−3^ and −4.0 × 10^−3^, respectively.

Fig 6 presents contour plots of PPA-*SHAD*, PA-*SEHD*, and DA-*H* models. Contour maps are projected onto the structures of compounds **5-3q**, **2-2b**, and **4-6b** for a straightforward association between the fields and different chemical structures. Color-coded isosurfaces represent regions in which the presence of a chemical group exhibiting the associated property (e.g., hydrophobic) contributes positively or negatively to the activity.

### PPA-SHAD model 3D-QSAR contours analysis

Fig 6A presents the steric *S* field of the PPA-*SHAD* model in green (favorable) and yellow (unfavorable) isosurfaces. A yellow contour at position 5 (R_3_) of the central pyridazinone ring of compound **5-3q** (denoted as *S*1 in Fig 6A) indicates that bulky groups at this position may disfavor the inhibitory activity. Most compounds have a methyl group at this location; however, the most active compound **5-3q** has R_3_ = H. Another yellow contour (denoted as *S*2 in Fig 6A) is located at position 5 (R_3’_) of the distal pyridazinone ring of compound **5-3q** indicating that smaller substituents are preferred at this position. This contour reflects that the most active compounds have the pyridazinone with H and methyl groups as R_3’_ substituents (with CH_3_ pointing downwards in Fig 6, *R* enantiomer). Less active compounds replace the pyridazinone ring by pyrazolone at this region (e.g., **5-3o**, **5-3v**, **5-3n**), where the latter uniquely features an additional CH_3_ instead of H at R_3’_ substituent position.

A great green contour which encompasses most of the phenoxyalkyl linker and part of the distal pyridazinone ring of compounds from set **5** (denoted as *S*3 in Fig 6A) indicates that bulk groups are desired in this region (compounds that possess such linker are the most active of the entire inhibitor series). Another yellow contour denoted as *S*4 in Fig 6A is in front of the position 3 of the pyrazolopyridine denoting that bulky substituents at this position are unfavorable for the activity. Indeed, the inhibitors from set **1** having the pyridazinone moiety at this location (Fig 1A) are the most inactive compounds. Another green contour denoted as *S*5 in Fig 6A is positioned at the amide nitrogen atom at position 2 of the inner pyridazinone ring of compound **5-3q**, which is the connecting point to the linker group in compounds of set **5** (Fig 1D). This contour is associated with the presence of the linker in the most active compounds in set **5**.

Fig 6A presents the hydrophobic *H* field of the PPA-*SHAD* model in olive-green (favorable) and white (unfavorable) isosurfaces. An olive green isosurface (denoted as *H*1 in Fig 6A) is observed at positions 4 and 5 of the central pyridazinone moiety, which indicates that the most hydrophobic pyridazinone group is preferred instead of pyrazolone in this region. Another olive-green contour (denoted as *H*2 in Fig 6A) at position 2 of the pyrazolopyridine indicates that hydrophobic groups are the best option here. A great white contour at the distal pyridazinone ring (denoted as *H*3 in Fig 6A) shows that hydrophilic groups are desired in this region, which is the characteristic of the most active compounds in set **5**. A smaller white isosurface (denoted as *H*4 in Fig 6A) over position 2 (R_1_) of the pyrazolopyridine suggests that less hydrophobic groups at this position favor the inhibitory activity. In fact, the presence of ethyl and CF_3_ at this position is preferred instead of isopropyl.

Fig 6B presents the H-bond acceptor *A* field of the PPA-*SHAD* model in brown (favorable) and magenta (unfavorable) isosurfaces. A brown contour (denoted as *A*1 in Fig 6B), located over the carbonyl group of the central pyridazinone ring, indicates that an H-bond acceptor group is the best option at this position. The carbonyl group at this position is a fundamental part of the scaffold of the studied PDE3A inhibitors; its 3D position slightly changes when pyrazolone replaces pyridazinone. A small brown contour (denoted as *A*2 in Fig 6B) is at position 7 of the pyrazolopyridine; most of the more active compounds (the ones from the set **5**) have a methoxy group at this location. A magenta isosurface (denoted as *A*3 in Fig 6B) is in front of the oxygen atom of the phenoxyalkyl linker of compound **5-3q**, indicating that H-bond acceptors at this region decrease the activity. This contour is also located in front of the polar atoms of the pyridazinone from compounds of set **1** (check the alignment in Fig 4B); therefore, the presence of this contour could be related to the decrease of the activity in compounds that have the pyridazinone at the pyrazolopyridine position 3. Another magenta isosurface (denoted as *A*4 in Fig 6B) near the carbonyl group of the distal pyridazinone indicates that H-bond acceptors are not desired in this zone. This region is occupied by the carbonyl group of the compounds from set **5** which replace the pyridazinone ring by pyrazolone (i.e., **5-3o**, **5-3v**, **5-3n**, **5-3w**, **5-3j**, **5-3m**), and are less active within the series.

Fig 6B shows the H-bond donor *D* field of the PPA-*SHAD* model in purple (favorable) and cyan (unfavorable) isosurfaces. A purple isosurface (denoted as *D*1 in Fig 6B) is at position 2 of the central pyridazinone ring of compound **5-3q**, which may be related to the presence of a protonated amine group in compounds from sets **2−4** that is not present in the less active compounds from the set **1**. A cyan isosurface (denoted as *D*2 in Fig 6B) near the position 1 of the pyrazolopyridine indicates that H-bond donors are unfavorable in this region. Several low active compounds of the sets **2** and **4** have substituents at position 7 of the pyrazolopyridine (R_2_) that contains H-bond donor groups in this region. Another cyan isosurface (denoted as *D*3 in Fig 6B) is placed at the protonated amine of the distal pyridazinone. This contour contradicts the binding modes observed in the docking experiments since this amino group acts as an H-bond donor in interactions of the most active compounds (set **5**) with the residue Gln1001 (Fig 3E).

### PA-SEHD model 3D-QSAR contours analysis

The FQSAR study applied to the PPA scheme considers that the pyrazolopyridine is the common core of the whole set; thereby, it did not contemplate the ligand binding behavior of the studied PDE3A inhibitors. The PA scheme is more realistic because the common core is the pyridazinone, which contains the groups forming the frequent interactions with the target protein defined previously as ECIDALs. Fig 6C and 6D present the field contours for the best model using the PA scheme, PA-*SEHD*. Despite the obvious differences between PPA and PA schemes (Fig 4B and 4C), examination of steric and hydrophobic fields revealed that the resulting FQSAR models share some common features (Fig 6C) listed below:

- Steric yellow contour denoted as *S*1 in Fig 6C (in analogy to *S*1 in Fig 6A, PPA scheme) indicates that bulky groups at position 5 (R_3_) of the central pyridazinone ring of compound **5-3q** may disfavor the inhibitory activity.
- Steric yellow contour denoted as *S*2 in Fig 6C (in analogy to *S*2 in Fig 6A, PPA scheme) indicates that smaller substituents are preferred at position 5 (R_3’_) of the compound **5-3q** distal pyridazinone ring.
- Steric green contour denoted as *S*3 in Fig 6C covers a large portion of the central pyridazinone and the pyrazolopyridine of the set **5** compounds, indicating the importance of bulky groups in this region. There is another analogy here with the PPA scheme, where the big green contour *S*3 in Fig 6A shows that compounds which possess a linker are the most active of the entire inhibitor series.
- Hydrophobic contour denoted as *H*1 in Fig 6C (in analogy to *H*1 in Fig 6A, PPA scheme) indicates that the most hydrophobic pyridazinone group is preferred instead of pyrazolone in the region near the positions 4 and 5 of the central pyridazinone moiety.
- The olive-green hydrophobic contour denoted as *H*2 in Fig 6C (in analogy to *H*2 in Fig 6A, PPA scheme) indicates that hydrophobic groups are the best option at position 2 of the pyrazolopyridine.
- The white hydrophobic contour denoted as *H*3 in Fig 6C (in analogy to *H*3 in Fig 6A, PPA scheme) indicates that hydrophilic groups are desired at the distal pyridazinone ring region.
- The white hydrophobic contour denoted as *H*4 in Fig 6C (in analogy to *H*4 in Fig 6A, PPA scheme) indicates that less hydrophobic groups at position 2 (R_1_) of the pyrazolopyridine favor the inhibitory activity.

There are three steric contours *S*4’, *S*5’ and *S*6’ present only in the PA scheme (Fig 6C). *S*4’ is a yellow isosurface located at the outer part of the substituents at position 7 (R_2_) of the pyrazolopyridine, which indicates the preference of smaller groups at this position. For instance, the compound **2–2l** having a phenyl group at this position is in the lower spectrum of activity. *S*5’ is a green isosurface located at position 2 (R_1_) of the pyrazolopyridine, which indicates that bulky groups are favored at this position for compounds in sets **1–4**. Indeed, the less active compounds from set **1**, the ones with pyridazinone analog substituents at position 3 of the pyrazolopyridine, do not occupy this region (see the alignment in Fig 4C). *S*6’ is a green isosurface located at position 5 (R_3_) of the pyrazolopyridine of compounds in sets **1–4**, which indicates that a bulky group at this position is beneficial for the activity. Certainly, R_3_ = CH_3_ is common among the studied compounds, where those lacking such group are usually less active than their counterparts.

Regarding the H-bond donor field, only the *D*2 cyan contour is typical for PPA and PA schemes (Fig 6D). This contour indicates that H-bond donors are unfavorable near the position 1 of the pyrazolopyridine. Another cyan contour (denoted as *D*1’ in Fig 6D) in front of the position 3 of the pyrazolopyridine moiety indicates that an H-bond donor group is not a good option at this position. For instance, compounds **3–6** and **4-6p** with benzimidazole replacing the pyrazolopyridine contain the NH of the imidazole ring at this position, with a decrease of the PDE3A inhibitory activity.

In the PA scheme, the H-bond acceptor field did not contribute to the FQSAR model, but instead, the electrostatic *E* field has a significant contribution. The *E* field of the PA-*SEHD* model is presented in Fig 6D, where blue and red contours indicate regions where positive and negative charge densities are respectively favorable for the activity.

A blue isosurface (denoted as *E*1 in Fig 6D) is in a region where there are different chemical groups in the sets under study. Compounds from set **5** have the phenoxyalkyl linker oxygen atom in this region, compounds from sets **2**–**4** have substituents at position 7 of the pyrazolopyridine, and compounds from set **1** have a high variability due to different orientations of the pyrazolopyridine moiety in the PA scheme. Contour *E*1 suggests that atoms with a more positive charge density are preferred at this position. A red contour (denoted as *E*2 in Fig 6D) at positions 1 and 2 of the pyrazolopyridine of compound **5-3q** indicates that negative charge densities at this position favor the inhibitory activity. This region is occupied by nitrogen atoms of the pyrazole ring (compounds from set **5**) when the number (*n*) of CH_2_ groups of the alkyl linker is *n* ≥ 4; compounds meeting this criterion are the most active within inhibitors from set **5**. Compounds from this set that have n < 4 change the 3D positions of the pyrazole nitrogen atoms (see the alignment in Fig 4C).

### DA-H model 3D-QSAR contours analysis

The FQSAR study applied to the DA scheme considers the active conformations obtained using docking experiments and their interactions with the surrounding residues in the PDE3A binding site. In this sense, the derived contours facilitate a meaningful interpretation of the structure-activity relationship of the studied compounds with their interactions inside the protein binding site. Fig 6E presents contours of the resulting DA-*H* model where the hydrophobic *H* field is in olive-green (favorable) and white (unfavorable) isosurfaces; compounds **2**-**2b** and **5-3q** are included to show the pyrazolopyridine moiety for compounds of sets **1–5**. Isosurfaces mentioned above, *H*2 (olive green) and *H*3 (white) are also found in the DA-*H* model, albeit they are larger considering the slight misalignment of the pyridazinone/pyrazolone and pyrazolopyridine rings observed in docking poses (Fig 3E). *H*2, occupied by substituents such as CF_3_ or methoxy groups at position 2 (R_1_) of the pyrazolopyridine for compounds sets **2** and **3,** and substituents at position 7 (R_2_) of the pyrazolopyridine for compounds of set **4**, is surrounded by the hydrophobic side chains of residues Tyr749, Leu908, Ile951 and Ile968 of PDE3A. This contour reflects that hydrophobic groups are favored inside this pocket. In contrast, *H*3 is surrounded by the side chains of Tyr749 (hydroxyl group), His961, Thr965 (hydroxyl group), and Gln1001, which contain polar groups; therefore, it is reasonable the presence of the unfavorable effect of hydrophobic groups in this region to the PDE inhibitory activity.

Another olive-green *H* contour denoted as *H*1” in this manuscript is observed near the hydrophobic residues Phe972, Leu1000, and Phe1004 (Fig 6E). Different hydrophobic chemical features from compounds of the sets **1**–**5** are placed in this region, suggesting that the presence of hydrophobic groups there is essential for the inhibitory activity, including aromatic groups forming the π-stacking with Phe1004.

Another white contour, denoted as *H*4” in this manuscript, is found near the contour *H*1”, towards the external surface of the protein. This contour indicates that hydrophilic groups are preferred in this region; however, no evident interaction is observed since this region of the active site is solvent exposed. For instance, several oxygen-containing chemical groups at position 7 of the most active compounds from set **2** are placed in this region.

It is noticeable that the DA-*H* model shares some similarities with models PA-*SEHD* and PA-*SEHD*, albeit reveals less information about features that may be important for the inhibitory activity, since *S*, *A* and *D* fields did not contribute to the FQSAR model applied on DA scheme. Despite that, the *H* field examination inside the PDE3A active site 3D structure helped to get a connection between the structure-activity relationship of the studied sets and the pockets defined by the residues in this protein for identifying key chemical properties that may aid in the design of novel and more potent PDE3A inhibitors.

## Conclusions

Binding orientations and structure-activity relationship of bicyclic heteroaromatic pyridazinones and pyrazolones as PDE3A inhibitors were studied by using molecular docking and field-based 3D-QSAR methods. A PDE3A homology model was constructed due to the lack of its crystallographic data. Docking of the studied compounds inside this model reproduced structural features reported for other PDEs. We found that most compounds exhibited similar orientations and interactions within the active site, where the pyridazinone/pyrazolone ring formed H-bond with His961 and Gln1001, and a π-stacking interaction with Phe1004; these interactions were defined as ECIDALs in the docking experiments previously mentioned. Additionally, the remaining inhibitors groups mainly displayed hydrophobic interactions with active site residues; these interactions explain the differential activities.

Different molecular alignment strategies, template and docking alignments, were used for the construction of the FQSAR models. Models with adequate statistical significance and acceptable prediction power were obtained using all the alignment strategies. Despite the distinct relative orientations obtained from the different alignments, common structural characteristics were identified for the developed FQSAR models, where steric and hydrophobic features seem to be the main factors influencing the differential inhibitory potency. The model applied to the docking alignment scheme enabled the analysis between QSAR contours and the regions in the binding site.

Overall, the information reported here will be useful for further optimization of PDE3A inhibitors.

## Supporting information

S1 File Structures and activities of the studied PDE3A inhibitors, information about the distribution of the activity values, equilibration of MD simulations, and residual plots between predicted and experimental values for the FQSAR models. (DOCX)
