# Supplementary material for: Docking and quantitative structure–activity relationship of bi-cyclic heteroaromatic pyridazinone and pyrazolone derivatives as phosphodiesterase 3A (PDE3A) inhibitors
Source: PLoS One. 2017 Dec 7;12(12):e0189213. doi: 10.1371/journal.pone.0189213 (PMC5720733; doi:10.1371/journal.pone.0189213)
Supplement: S1 File — (DOCX) [file pone.0189213.s001.docx]

S1 File. Structures and activities of the studied PDE3A inhibitors, information about the dis-tribution of the activity values, equilibration of MD simulations, and residual plots be-tween predicted and experimental values for the FQSAR models.

**Supporting information for**

Docking and quantitative structure–activity relationship of bicyclic heteroaromatic pyridazinone and pyrazolone derivatives as phosphodiesterase 3A (PDE3A) inhibitors

Camila Muñoz-Gutiérrez^1^, Daniela Cáceres-Rojas^1^, Francisco Adasme-Carreño^1^, Iván Palomo^2^, Eduardo Fuentes^2,3^, Julio Caballero^1^*.

^1^ Centro de Bioinformática y Simulación Molecular (CBSM), Universidad de Talca, Talca, Chile.

^2^ Platelet Research Laboratory, Department of Clinical Biochemistry and Immunohematology, Faculty of Health Sciences, Interdisciplinary Excellence Research Program on Healthy Aging (PIEI-ES), Universidad de Talca, Talca, Chile.

^3^ Núcleo Científico Multidisciplinario, Universidad de Talca, Talca, Chile.

# Fig A. Histogram of the activity values. Activities were rounded to multiples of 0.5.


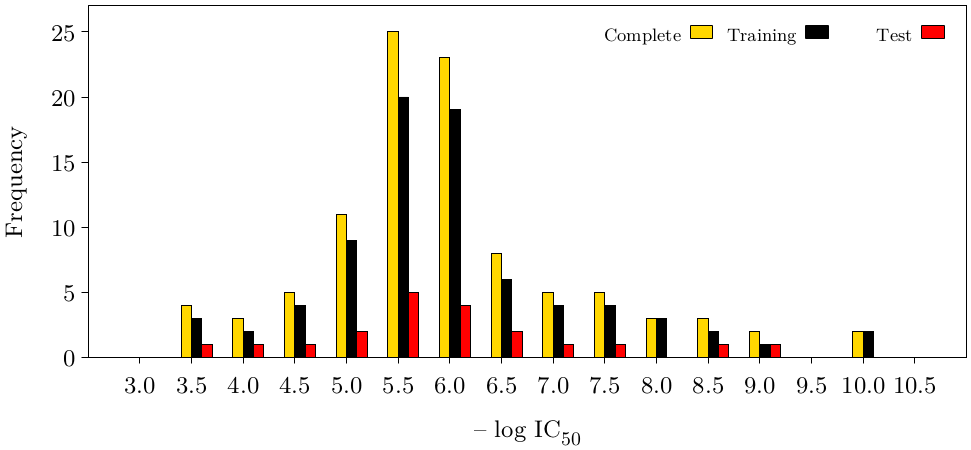


**Fig B.** **Fluctuation of molecular structures during equilibration MD simulation.** Root mean square deviation (RMSD, in Å) was measured along the MD trajectory for protein heavy atoms (black), ligand heavy atoms (green), magnesium ions (pink), and metal coordination waters (red).


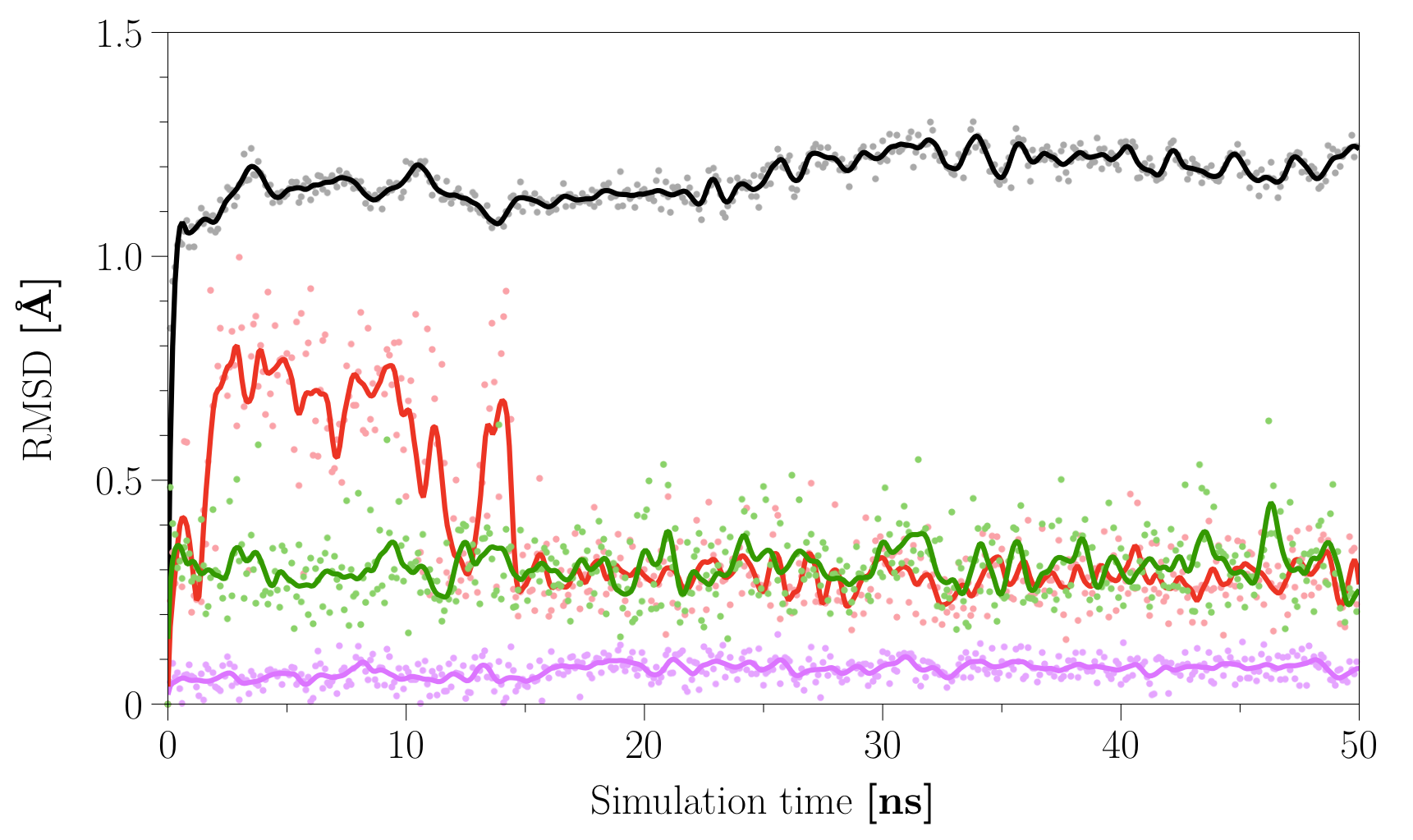


**Fig C.** **Fluctuation of protein-ligand intermolecular interactions during equilibration MD simulation.** Distances (in Å) were measured along the MD trajectory for H-bonds established between ligand and residues His961 (light blue), Gln1001-CO (brown), and Gln1001-NH_2_ (light brown); and π-stacking interaction with Phe1004 (purple). H-bond distances are measured between heavy (donor and acceptor) atoms.


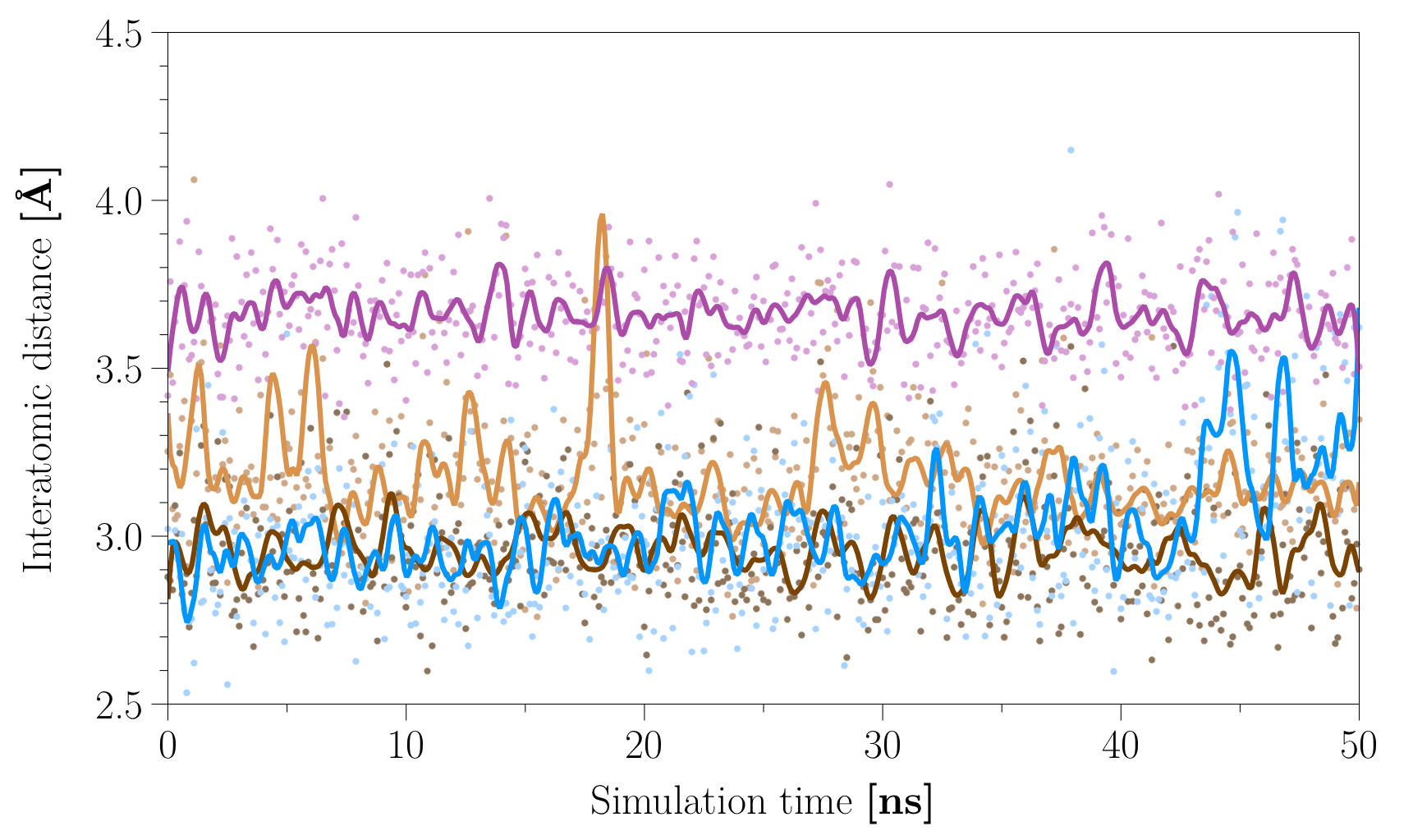


**Fig D**. **Residual plots between predicted and experimental values for the three FQSAR models based on the pyrazolopyridine (PPA), pyridazinone/pyrazolone (PA), and docking (DA) alignments.**


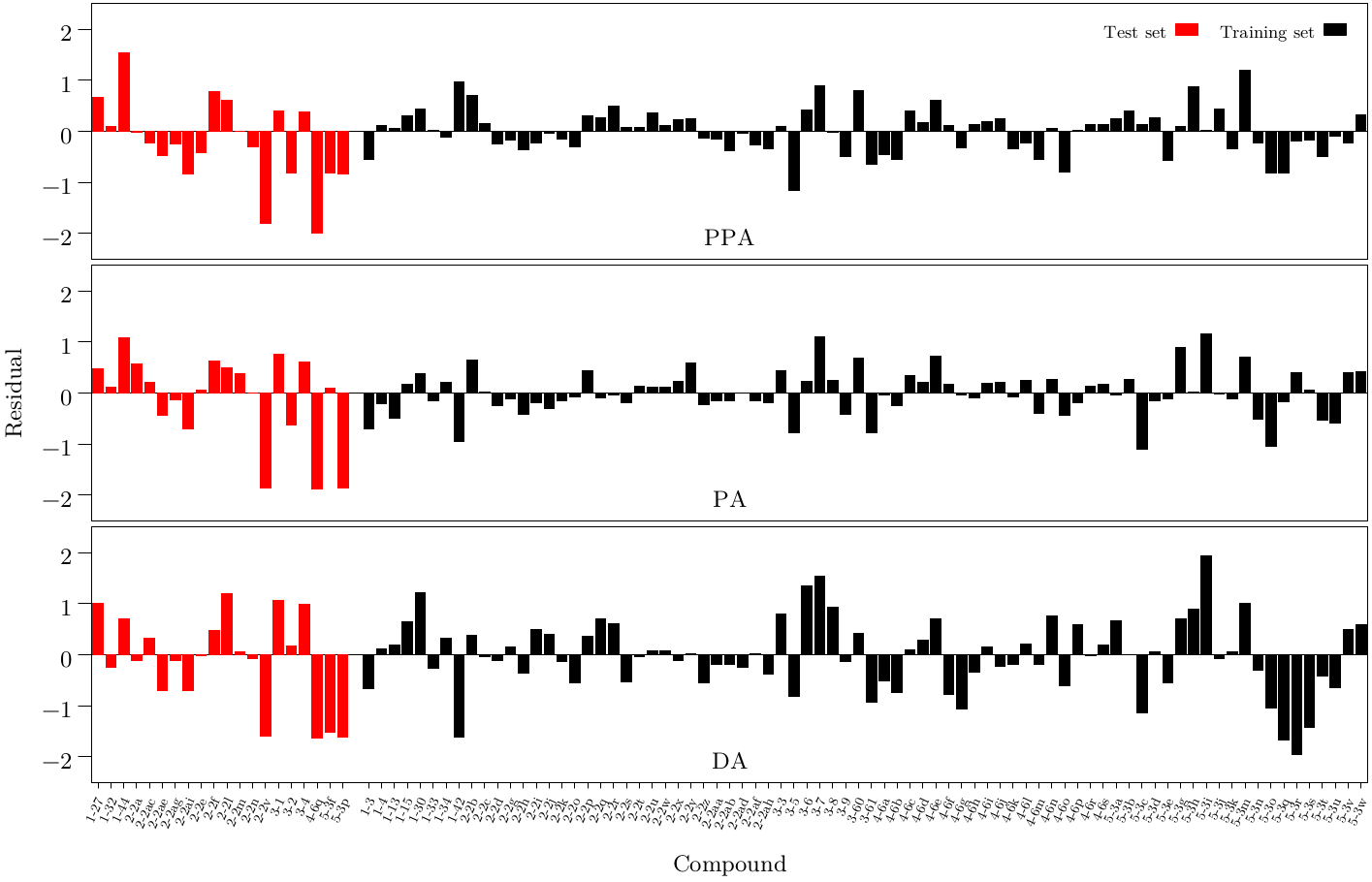


**Table A**. **Structures and activities of the studied PDE3A inhibitors.** Experimental and predicted activities log(10^6^/IC_50_) (in µM) using models FQSAR models. *^a^*

| **Ligand** | **QSAR set** | **Structure** | | | | | | | | | | **activity** | | | |
| --- | --- | --- | --- | --- | --- | --- | --- | --- | --- | --- | --- | --- | --- | --- | --- |
|  |  |  |  |  |  |  |  |  |  |  |  | **Exp.** | **PPA** | **PA** | **DA** |
| 1-2 | train | 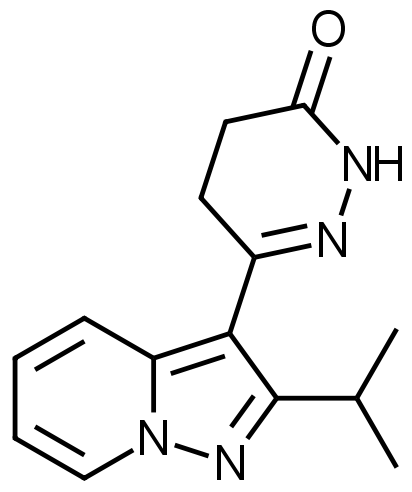 | | | | | | | | | | 4.000 | 5.216 | 4.662 | 5.341 |
| 1-3 | train | 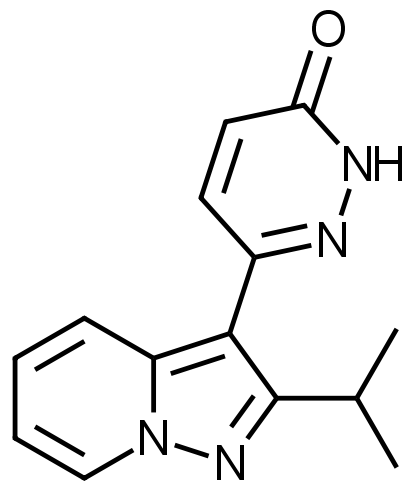 | | | | | | | | | | 4.143 | 5.124 | 5.002 | 5.462 |
| 1-4 | train | 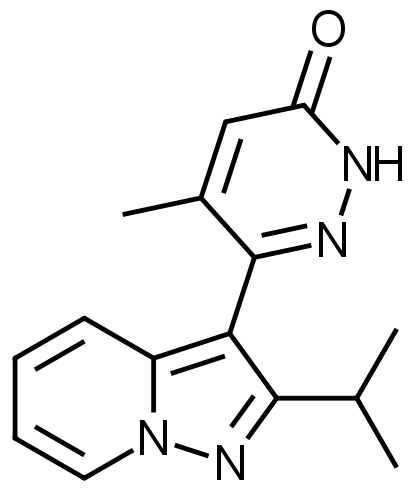 | | | | | | | | | | 3.398 | 3.699 | 4.294 | 4.591 |
| 1-13 | test | 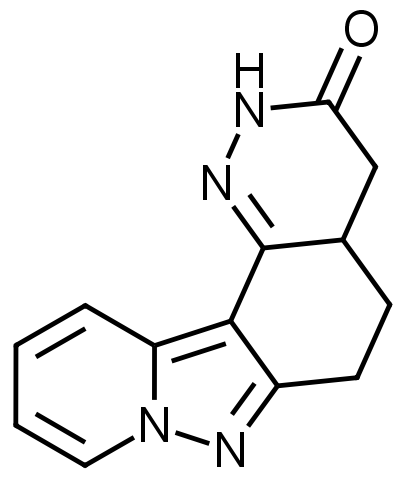 | | | | | | | | | | 5.155 | 4.350 | 4.154 | 4.678 |
| 1-14 | - | 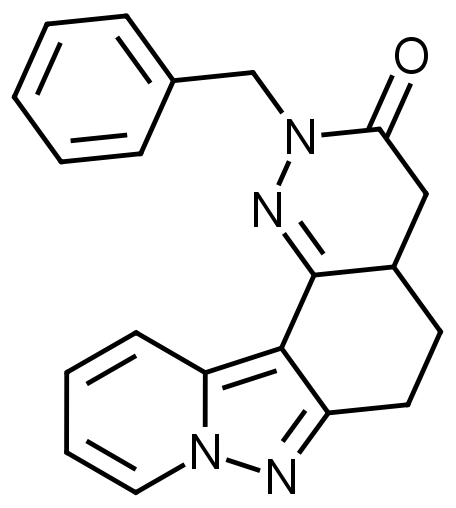 | | | | | | | | | | 4.585 | - | - | - |
| 1-15 | train | 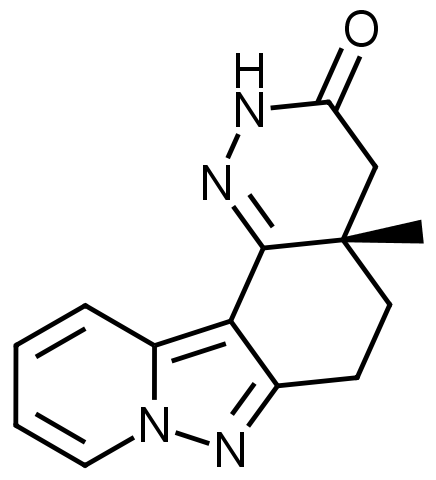 | | | | | | | | | | 4.824 | 3.585 | 3.449 | 3.488 |
| 1-16*^b^* | - | 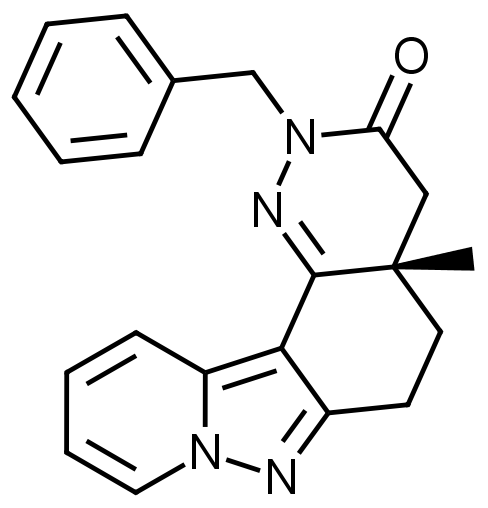 | | | | | | | | | | 5.051 | - | - | - |
| 1-17*^b^* | - | 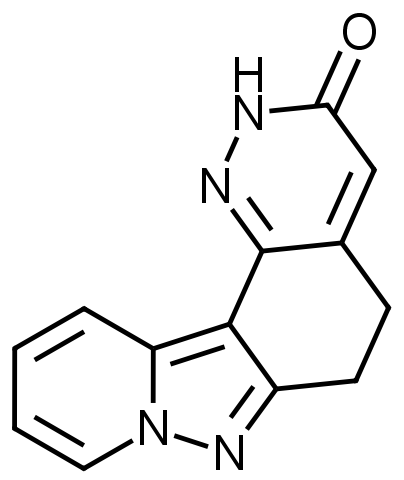 | | | | | | | | | | 5.495 | - | - | - |
| 1-18*^b^* | - | 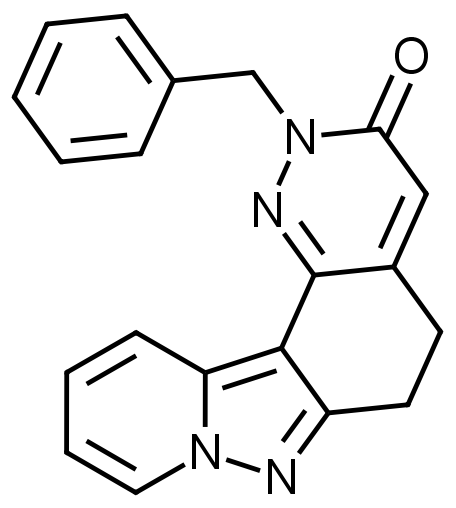 | | | | | | | | | | 4.161 | - | - | - |
| 1-27 | train | 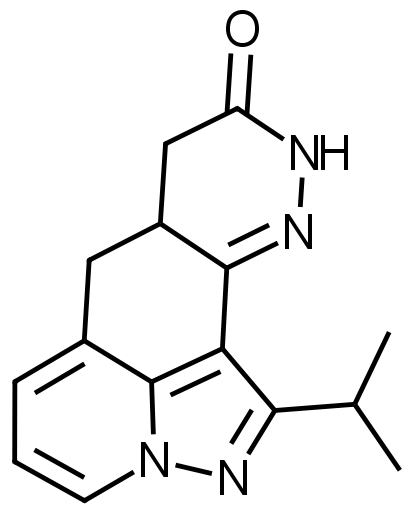 | | | | | | | | | | 3.678 | 4.026 | 3.973 | 4.809 |
| 1-30 | test | 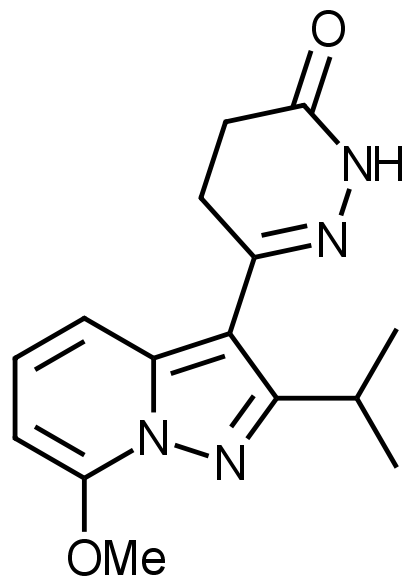 | | | | | | | | | | 3.585 | 3.491 | 3.510 | 3.158 |
| 1-31*^b^* | - | 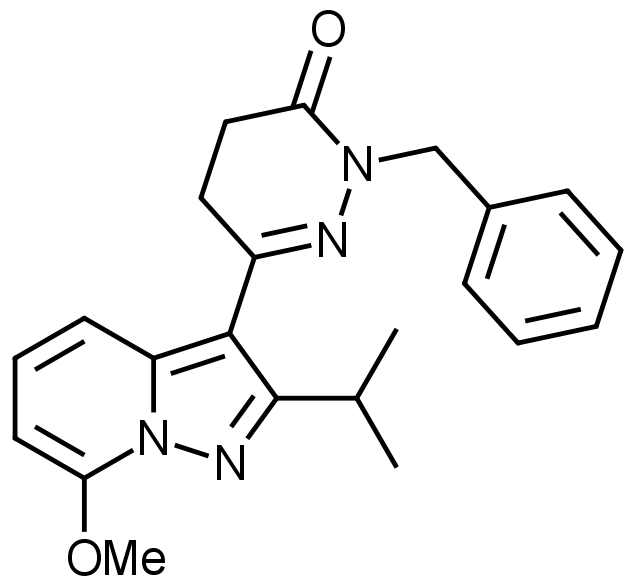 | | | | | | | | | | 3.398 | - | - | - |
| 1-32 | train | 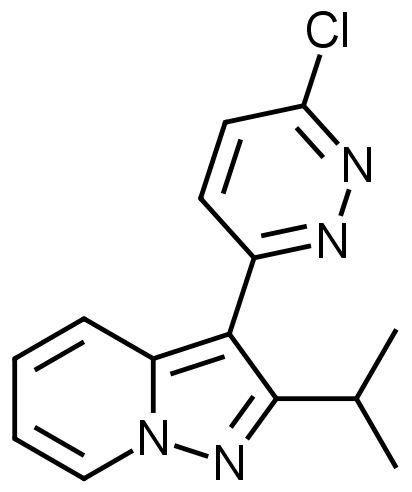 | | | | | | | | | | 3.398 | 3.408 | 3.251 | 3.136 |
| 1-33 | train | 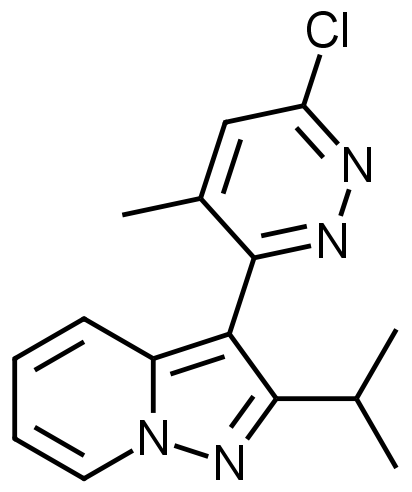 | | | | | | | | | | 3.398 | 3.279 | 3.607 | 3.715 |
| 1-34 | train | 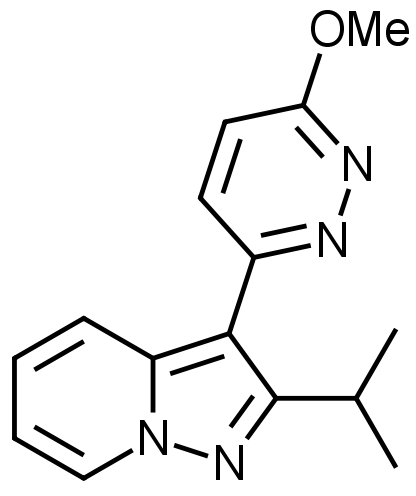 | | | | | | | | | | 3.398 | 3.516 | 3.190 | 3.517 |
| 1-42 | train | 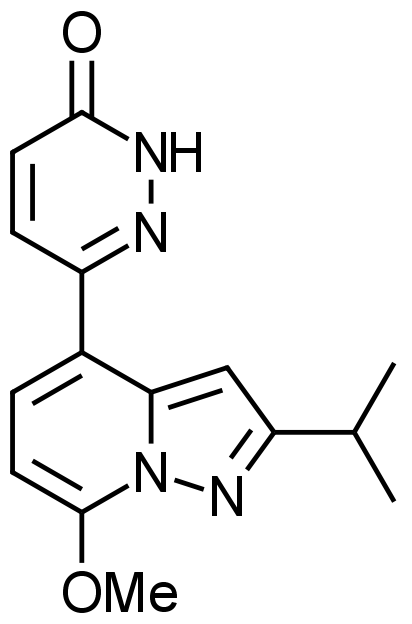 | | | | | | | | | | 5.561 | 6.529 | 4.612 | 3.938 |
| 1-43*^b^* | - | 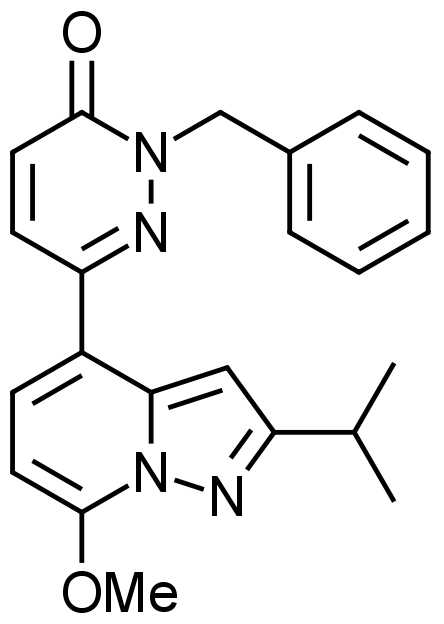 | | | | | | | | | | 4.382 | - | - | - |
| 1-44 | test | 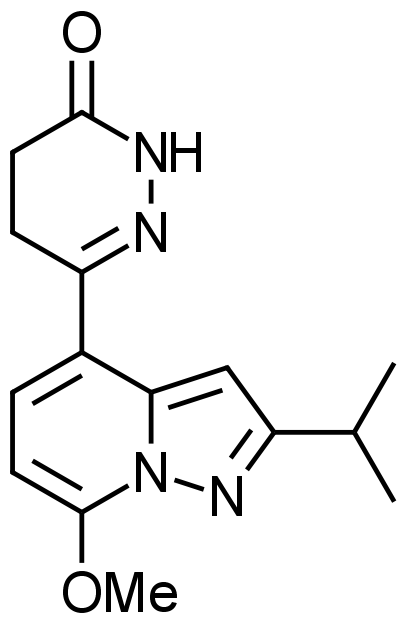 | | | | | | | | | | 4.402 | 5.946 | 5.476 | 5.104 |
| 1-45*^b^* | - | 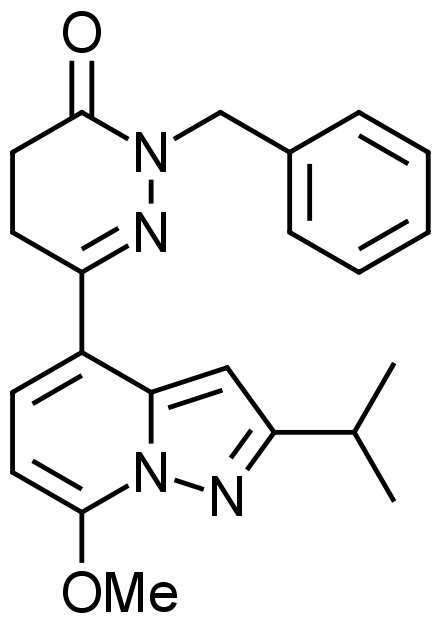 | | | | | | | | | | 4.475 | - | - | - |
| 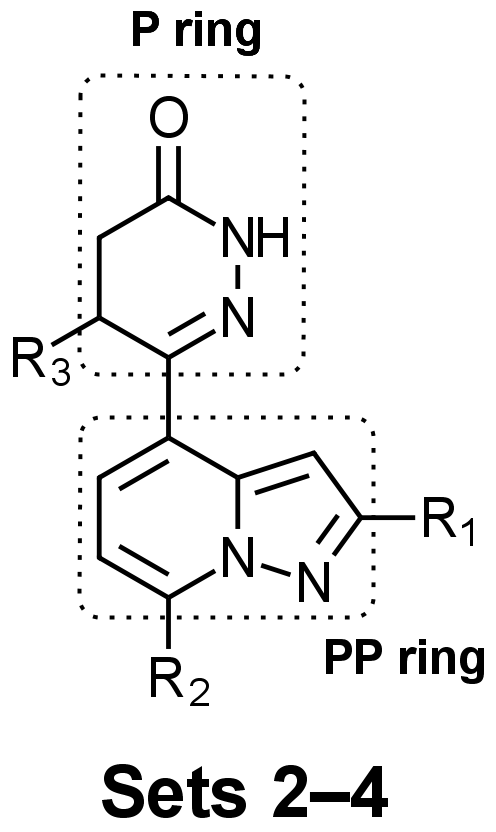 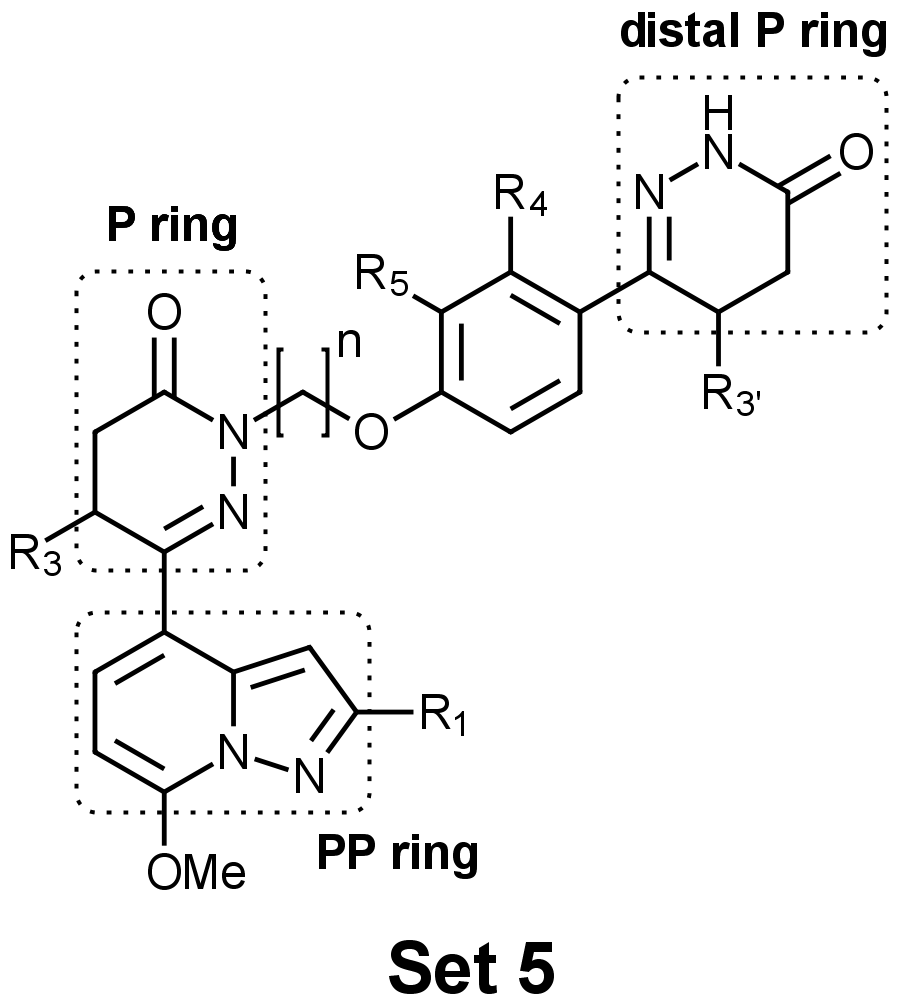 | | | | | | | | | | | | | | | |
| **Ligand** | **QSAR set** | **rings** | | | **R_1_** | **R_2_** | **R_3_** | **n** | **R_3’_** | **R_4_** | **R_5_** | **activity** | | | |
|  |  | **PP** | **P** | **distal P** |  |  |  |  |  |  |  | **Exp.** | **PPA** | **PA** | **DA** |
| 1-42 | train | 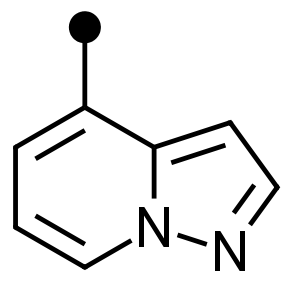 | 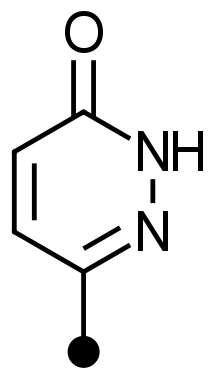 | - | i-Pr | OMe | H | - | - | - | - | 5.561 | 6.529 | 4.612 | 3.938 |
| 1-44 | test | 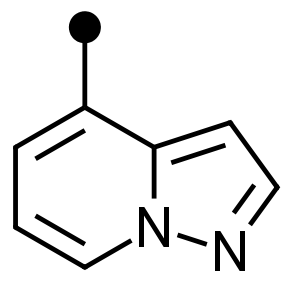 | 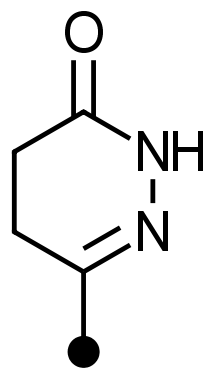 | - | i-Pr | OMe | H | - | - | - | - | 4.402 | 5.946 | 5.476 | 5.104 |
| 2-2a | test | 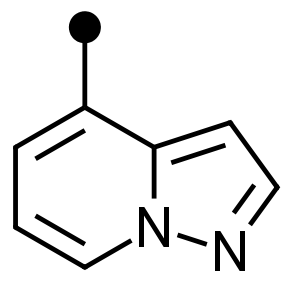 | 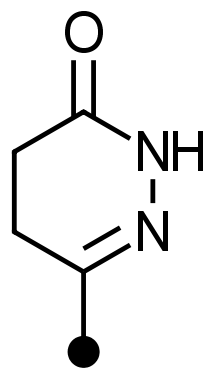 | - | Et | H | Me | - | - | - | - | 5.320 | 5.307 | 5.883 | 5.207 |
| 2-2b | train | " | " | - | Et | OMe | Me | - | - | - | - | 4.947 | 5.657 | 5.591 | 5.334 |
| 2-2c | train | " | " | - | Et | OEt | Me | - | - | - | - | 5.381 | 5.532 | 5.409 | 5.352 |
| 2-2d | train | " | " | - | Et | SMe | Me | - | - | - | - | 5.701 | 5.448 | 5.463 | 5.592 |
| 2-2e | test | " | " | - | Et | SOMe | Me | - | - | - | - | 5.614 | 5.196 | 5.670 | 5.591 |
| 2-2f | test | " | " | - | Et | SO_2_Me | Me | - | - | - | - | 5.147 | 5.919 | 5.778 | 5.621 |
| 2-2g | train | " | " | - | Et | NHMe | Me | - | - | - | - | 5.167 | 4.990 | 5.046 | 5.316 |
| 2-2h | train | " | " | - | Et | NMe_2_ | Me | - | - | - | - | 5.620 | 5.262 | 5.208 | 5.250 |
| 2-2i | train | " | " | - | Et | NH(CH_2_)_2_OH | Me | - | - | - | - | 5.013 | 4.791 | 4.828 | 5.508 |
| 2-2j | train | " | " | - | Et | morpholin-1-yl | Me | - | - | - | - | 5.045 | 5.001 | 4.745 | 5.449 |
| 2-2k | train | " | " | - | Et | NHAc | Me | - | - | - | - | 5.485 | 5.327 | 5.327 | 5.356 |
| 2-2l | test | " | " | - | Et | N(Me)Ph | Me | - | - | - | - | 4.776 | 5.391 | 5.272 | 5.976 |
| 2-2m | test | " | " | - | Et | Me | Me | - | - | - | - | 5.413 | 5.422 | 5.801 | 5.479 |
| 2-2n | test | " | " | - | Et | CH(F)Me | Me | - | - | - | - | 5.742 | 5.446 | 5.747 | 5.667 |
| 2-2o | train | " | " | - | Et | CH(OH)Me | Me | - | - | - | - | 6.041 | 5.737 | 5.961 | 5.483 |
| 2-2p | train | " | " | - | Et | C(OH)Me_2_ | Me | - | - | - | - | 5.289 | 5.600 | 5.721 | 5.651 |
| 2-2q | train | " | " | - | Et | Ph | Me | - | - | - | - | 5.132 | 5.392 | 5.046 | 5.834 |
| 2-2r | train | " | " | - | Et | CO_2_H | Me | - | - | - | - | 4.725 | 5.226 | 4.693 | 5.340 |
| 2-2s | train | " | " | - | Et | CONH_2_ | Me | - | - | - | - | 5.987 | 6.056 | 5.799 | 5.447 |
| 2-2t | train | " | " | - | Et | CN | Me | - | - | - | - | 5.587 | 5.660 | 5.728 | 5.550 |
| 2-2u | train | " | " | - | Et | Ac | Me | - | - | - | - | 5.614 | 5.980 | 5.730 | 5.696 |
| 2-2v | test | " | " | - | H | OMe | Me | - | - | - | - | 7.222 | 5.415 | 5.351 | 5.634 |
| 2-2w | train | " | " | - | Me | OMe | Me | - | - | - | - | 5.359 | 5.467 | 5.482 | 5.436 |
| 2-2x | train | " | " | - | Pr | OMe | Me | - | - | - | - | 5.408 | 5.639 | 5.644 | 5.300 |
| 2-2y | train | " | " | - | i-Pr | OMe | Me | - | - | - | - | 5.182 | 5.428 | 5.770 | 5.204 |
| 2-2z | train | " | " | - | c-Pr | OMe | Me | - | - | - | - | 5.889 | 5.765 | 5.659 | 5.332 |
| 2-2aa | train | " | " | - | CH_2_F | OMe | Me | - | - | - | - | 5.646 | 5.494 | 5.487 | 5.459 |
| 2-2ab | train | " | " | - | CHF_2_ | OMe | Me | - | - | - | - | 5.764 | 5.381 | 5.616 | 5.575 |
| 2-2ac | test | " | " | - | CF_3_ | OMe | Me | - | - | - | - | 5.623 | 5.402 | 5.840 | 5.955 |
| 2-2ad | train | " | " | - | CHFMe | OMe | Me | - | - | - | - | 5.656 | 5.614 | 5.657 | 5.402 |
| 2-2ae | test | " | " | - | CHCH_2_ | OMe | Me | - | - | - | - | 6.032 | 5.564 | 5.588 | 5.319 |
| 2-2af | train | " | " | - | CH_2_OH | OMe | Me | - | - | - | - | 5.373 | 5.110 | 5.212 | 5.394 |
| 2-2ag | test | " | " | - | CH_2_OMe | OMe | Me | - | - | - | - | 5.479 | 5.240 | 5.354 | 5.366 |
| 2-2ah | train | " | " | - | Ac | OMe | Me | - | - | - | - | 5.903 | 5.562 | 5.706 | 5.530 |
| 2-2ai | test | " | " | - | CN | OMe | Me | - | - | - | - | 6.301 | 5.465 | 5.597 | 5.589 |
| 3-1 | test | 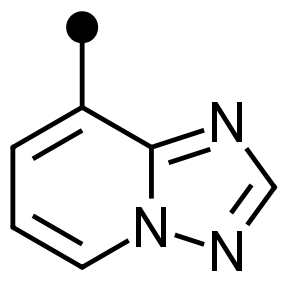 | " | - | CF_3_ | OMe | Me | - | - | - | - | 4.991 | 5.386 | 5.751 | 6.056 |
| 3-2 | test | 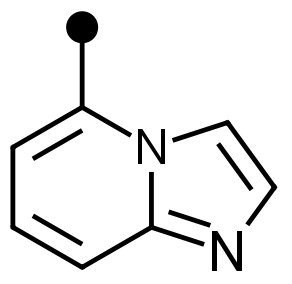 | " | - | CF_3_ | OMe | Me | - | - | - | - | 5.839 | 5.027 | 5.215 | 6.018 |
| 3-3 | train | 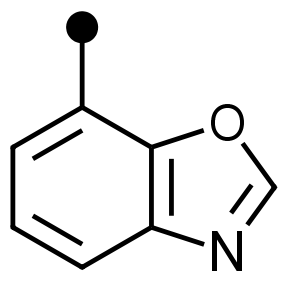 | " | - | CF_3_ | OMe | Me | - | - | - | - | 5.241 | 5.346 | 5.688 | 6.041 |
| 3-4 | test | 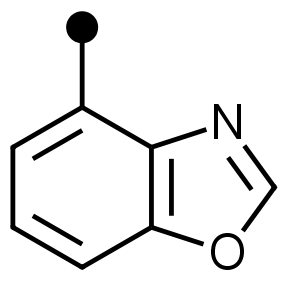 | " | - | CF_3_ | OMe | Me | - | - | - | - | 5.192 | 5.566 | 5.794 | 6.181 |
| 3-5 | train | 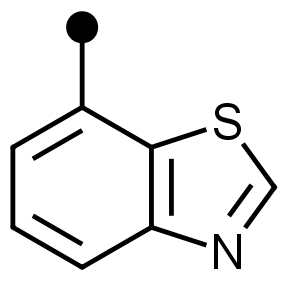 | " | - | CF_3_ | OMe | Me | - | - | - | - | 6.824 | 5.669 | 6.048 | 6.011 |
| 3-6 | train | 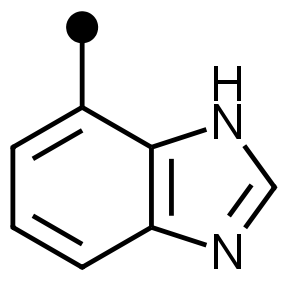 | " | - | CF_3_ | OMe | Me | - | - | - | - | 4.654 | 5.075 | 4.878 | 6.003 |
| 3-7 | train | 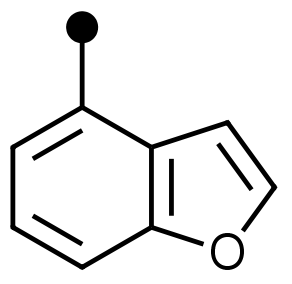 | " | - | CF_3_ | OMe | Me | - | - | - | - | 4.703 | 5.478 | 5.373 | 5.094 |
| 3-8 | train | 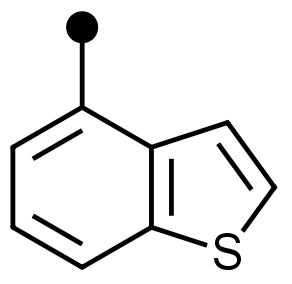 | " | - | CF_3_ | OMe | Me | - | - | - | - | 5.545 | 5.686 | 5.556 | 5.394 |
| 3-9 | train | 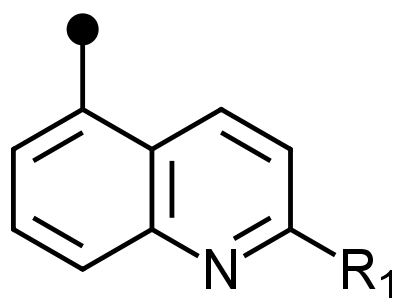 | " | - | CF_3_ | OMe | Me | - | - | - | - | 6.013 | 5.592 | 5.809 | 6.232 |
| 3-60 | train | 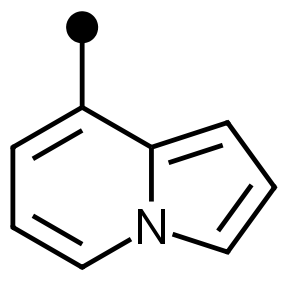 | " | - | Et | OMe | Me | - | - | - | - | 4.684 | 5.528 | 5.784 | 6.472 |
| 3-61 | train | 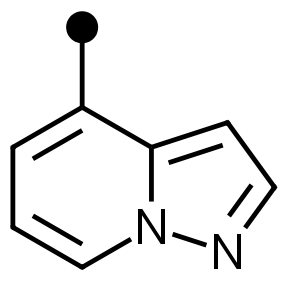 | " | - | Et | OMe | Me | - | - | - | - | 6.328 | 5.520 | 5.601 | 5.889 |
| 4-6a | train | " | 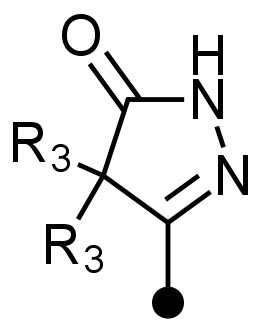 | - | Et | Me | Me | - | - | - | - | 5.625 | 5.172 | 5.589 | 5.112 |
| 4-6b | train | " | " | - | Et | Et | Me | - | - | - | - | 5.699 | 5.158 | 5.447 | 4.948 |
| 4-6c | train | " | " | - | Et | OMe | Me | - | - | - | - | 5.036 | 5.430 | 5.372 | 5.121 |
| 4-6d | train | " | " | - | Et | SMe | Me | - | - | - | - | 5.045 | 5.209 | 5.247 | 5.328 |
| 4-6e | train | " | " | - | Et | NHMe | Me | - | - | - | - | 4.122 | 4.733 | 4.848 | 4.833 |
| 4-6f | train | " | " | - | Et | CH_2_OH | Me | - | - | - | - | 5.799 | 5.905 | 5.965 | 5.023 |
| 4-6g | train | " | " | - | Et | CH(OH)Me | Me | - | - | - | - | 5.788 | 5.465 | 5.748 | 4.717 |
| 4-6h | train | " | " | - | Et | Ac | Me | - | - | - | - | 5.602 | 5.731 | 5.510 | 5.260 |
| 4-6i | train | " | " | - | H | OMe | Me | - | - | - | - | 4.965 | 5.155 | 5.160 | 5.121 |
| 4-6j | train | " | " | - | c-Pr | OMe | Me | - | - | - | - | 5.210 | 5.452 | 5.411 | 4.976 |
| 4-6k | train | " | " | - | CHF_2_ | OMe | Me | - | - | - | - | 5.484 | 5.148 | 5.405 | 5.297 |
| 4-6l | train | " | " | - | CF_3_ | OMe | Me | - | - | - | - | 5.384 | 5.163 | 5.627 | 5.583 |
| 4-6m | train | 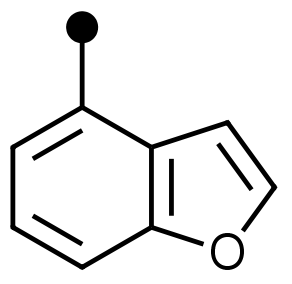 | " | - | CF_3_ | OMe | Me | - | - | - | - | 5.959 | 5.403 | 5.564 | 5.763 |
| 4-6n | train | 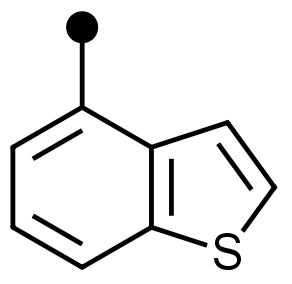 | " | - | CF_3_ | OMe | Me | - | - | - | - | 5.271 | 5.336 | 5.544 | 6.031 |
| 4-6o | train | 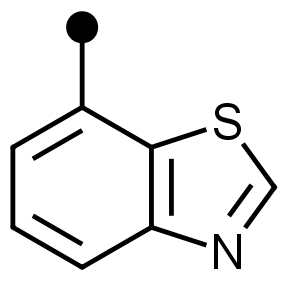 | " | - | CF_3_ | OMe | Me | - | - | - | - | 6.268 | 5.467 | 5.823 | 5.666 |
| 4-6p | train | 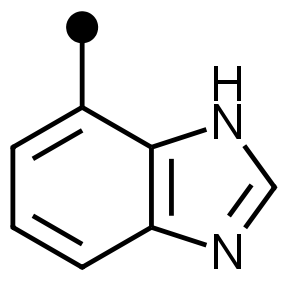 | " | - | CF_3_ | OMe | Me | - | - | - | - | 4.850 | 4.864 | 4.655 | 5.442 |
| 4-6q | test | 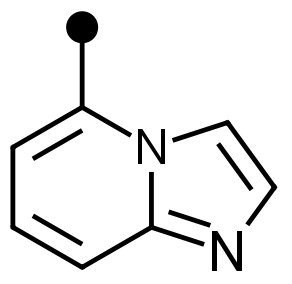 | " | - | CF_3_ | OMe | Me | - | - | - | - | 6.854 | 4.866 | 4.972 | 5.216 |
| 4-6r | train | 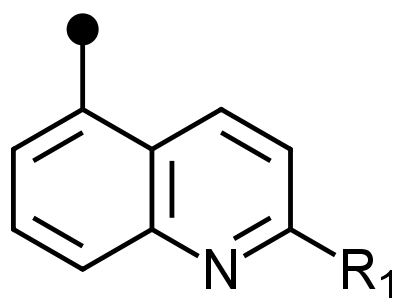 | " | - | Et | OMe | Me | - | - | - | - | 4.997 | 5.128 | 5.123 | 4.985 |
| 4-6s | train | " | " | - | CF_3_ | OMe | Me | - | - | - | - | 5.177 | 5.314 | 5.347 | 5.373 |
| 5-3a | train | 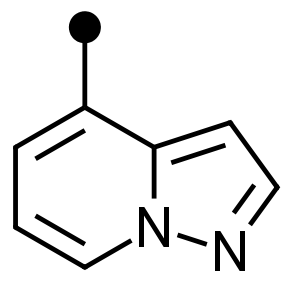 | 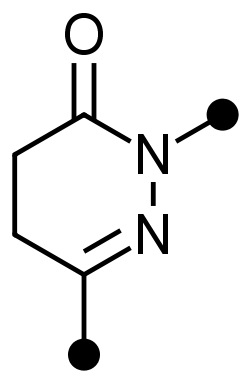 | 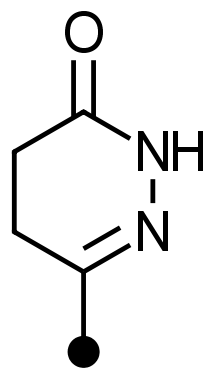 | CF_3_ | OMe | Me | 2 | Me | H | H | 6.886 | 7.127 | 6.857 | 7.554 |
| 5-3b | train | " | " | " | CF_3_ | OMe | Me | 3 | Me | H | H | 6.854 | 7.244 | 7.126 | 6.863 |
| 5-3c | train | " | " | " | CF_3_ | OMe | Me | 4 | Me | H | H | 7.824 | 7.950 | 6.724 | 6.684 |
| 5-3d | train | " | " | " | CF_3_ | OMe | Me | 5 | Me | H | H | 7.143 | 7.410 | 6.996 | 7.200 |
| 5-3e | train | " | " | " | Et | OMe | Me | 3 | Me | H | H | 7.959 | 7.396 | 7.842 | 7.398 |
| 5-3f | test | " | " | " | Et | OMe | Me | 4 | Me | H | H | 8.921 | 8.105 | 9.010 | 7.395 |
| 5-3g | train | " | " | 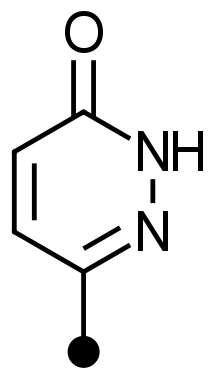 | CF_3_ | OMe | Me | 4 | Me | H | H | 4.398 | 4.493 | 5.294 | 5.104 |
| 5-3h | train | " | " | 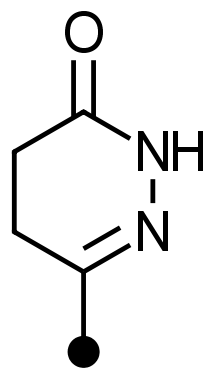 | CF_3_ | OMe | Me | 4 | H | H | H | 6.444 | 7.312 | 6.465 | 7.333 |
| 5-3i | train | " | " | 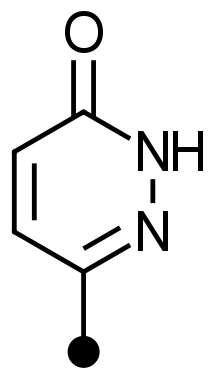 | CF_3_ | OMe | Me | 4 | H | H | H | 4.420 | 4.445 | 5.576 | 6.353 |
| 5-3j | train | " | " | 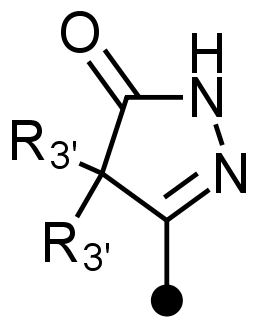 | CF_3_ | OMe | Me | 4 | Me | H | H | 6.569 | 7.006 | 6.559 | 6.499 |
| 5-3k*^c^* | train | " | " | 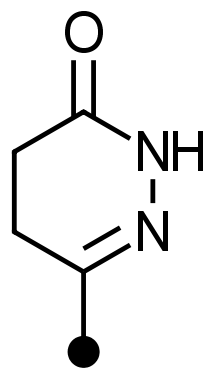 | CF_3_ | OMe | Me | 4 | Me | H | H | 6.229 | 5.880 | 6.120 | 6.279 |
| 5-3l*^bc^* | - | " | " | 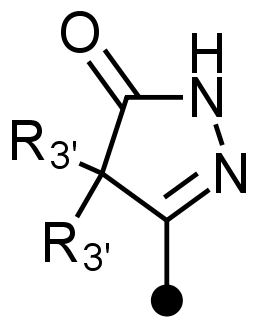 | CF_3_ | OMe | Me | 4 | Me | H | H | 5.762 | - | - | - |
| 5-3m | train | " | " | " | CF_3_ | OMe | Me | 4 | Me | H | F | 5.818 | 7.017 | 6.531 | 6.828 |
| 5-3n | train | " | " | " | CF_3_ | OMe | Me | 4 | Me | F | H | 7.237 | 7.001 | 6.720 | 6.939 |
| 5-3o | train | " | " | " | CF_3_ | OMe | Me | 4 | Me | F | F | 7.824 | 7.008 | 6.774 | 6.777 |
| 5-3p | test | " | " | 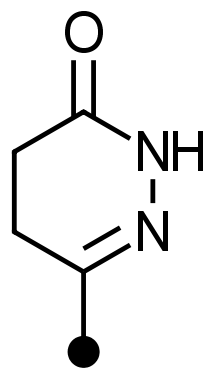 | CF_3_ | OMe | Me | 4 | H | F | F | 8.301 | 7.462 | 6.442 | 6.694 |
| 5-3q | train | " | " | " | Et | OMe | H | 4 | Me | H | H | 9.569 | 8.759 | 9.404 | 7.890 |
| 5-3r | train | " | 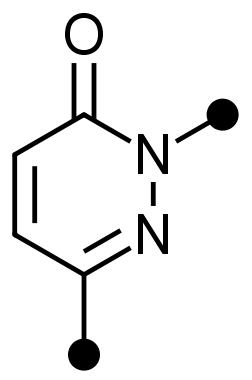 | " | Et | OMe | H | 4 | Me | H | H | 8.569 | 9.380 | 9.976 | 7.618 |
| 5-3s | train | " | 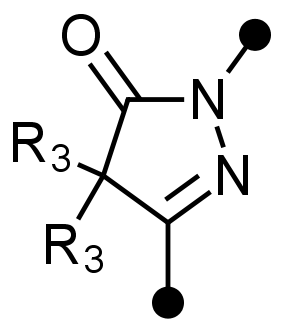 | " | Et | OMe | Me | 4 | Me | H | H | 8.770 | 8.600 | 8.828 | 7.343 |
| 5-3t | train | 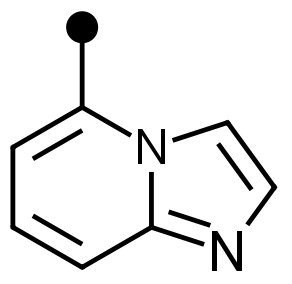 | " | " | CF_3_ | OMe | Me | 4 | H | F | F | 8.301 | 7.810 | 7.765 | 7.875 |
| 5-3u | train | 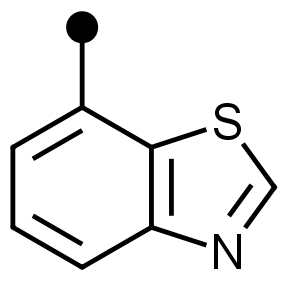 | " | " | CF_3_ | OMe | Me | 4 | H | F | F | 8.161 | 8.062 | 7.565 | 7.510 |
| 5-3v | train | 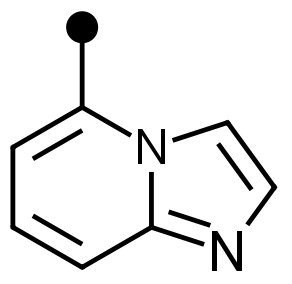 | " | 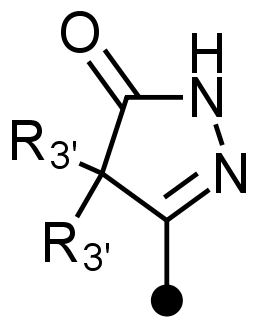 | CF_3_ | OMe | Me | 4 | Me | F | F | 7.398 | 7.177 | 7.788 | 7.890 |
| 5-3w | train | 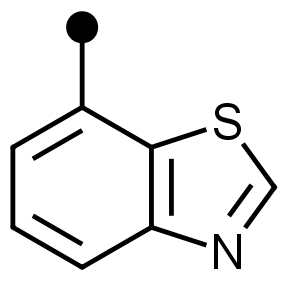 | " | " | CF_3_ | OMe | Me | 4 | Me | F | F | 7.201 | 7.514 | 7.622 | 7.785 |

*^a^* Ligand names indicate the source article as part number of the inhibitor series reported in references ^[14–18]^ and molecule name given within the corresponding article. Experimental and predicted activities (expressed as log(10^6^/IC_50_) obtained with FQSAR models for each alignment strategy (PPA = pyrazolopyridine alignment, PA = pyridazinone/pyrazolone alignment, DA = docking alignment) are shown. *^b^* Ligands which did not yield satisfactory docking poses, and were excluded from the FQSAR analysis. *^c^* Distal P group is at *meta* position instead of *para* position of the phenoxy group.
